# Toward Raman Subcellular Imaging of Endothelial Dysfunction

**DOI:** 10.1021/acs.jmedchem.1c00051

**Published:** 2021-04-06

**Authors:** Adriana Adamczyk, Ewelina Matuszyk, Basseem Radwan, Stefano Rocchetti, Stefan Chlopicki, Malgorzata Baranska

**Affiliations:** † Faculty of Chemistry, Jagiellonian University, 2 Gronostajowa Str., 30-387 Krakow, Poland; ‡ Jagiellonian Centre for Experimental Therapeutics (JCET), Jagiellonian University, 14 Bobrzynskiego Str., 30-348 Krakow, Poland; § Chair of Pharmacology, Jagiellonian University, 16 Grzegorzecka Str., 31-531 Krakow, Poland

## Abstract

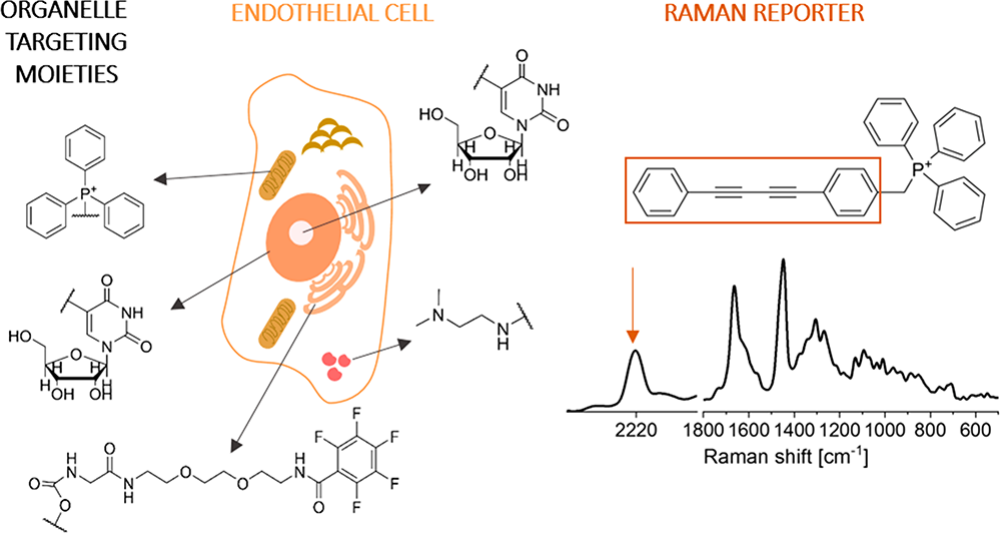

Multiple diseases are at some point associated with altered endothelial function, and endothelial dysfunction (ED) contributes to their pathophysiology. Biochemical changes of the dysfunctional endothelium are linked to various cellular organelles, including the mitochondria, endoplasmic reticulum, and nucleus, so organelle-specific insight is needed for better understanding of endothelial pathobiology. Raman imaging, which combines chemical specificity with microscopic resolution, has proved to be useful in detecting biochemical changes in ED at the cellular level. However, the detection of spectroscopic markers associated with specific cell organelles, while desirable, cannot easily be achieved by Raman imaging without labeling. This critical review summarizes the current advances in Raman-based analysis of ED, with a focus on a new approach involving molecular Raman reporters that could facilitate the study of biochemical changes in cellular organelles. Finally, imaging techniques based on both conventional spontaneous Raman scattering and the emerging technique of stimulated Raman scattering are discussed.

## 1 Introduction

Endothelial cells line the lumen of all the vessels in the body, from the heart to the capillaries,^1^ and can be regarded as the unique organ of the body that maintains cardiovascular homeostasis and accomplishes multiple roles by its endocrine, paracrine, and autocrine functions. The best studied is the endothelium-dependent regulation of vascular tone, performed through a fine-tuned balance between the activity of endothelial mediators with vasodilatory effects (*e.g.*, nitric oxide (NO), prostacyclin (PGI_2_), and epoxyeicosatrienoic acids (EETs)) or vasoconstricting effects (*e.g.*, endothelin-1 (ET-1), thromboxane A_2_ (TXA_2_), and angiotensin II (Ang II)).^1−3^ The endothelium regulates not only blood flow but also vascular permeability, adhesion of platelets and leukocytes to the endothelium, proliferation of smooth muscle cells, immune and inflammatory response, thrombotic processes, and angiogenesis. The mechanisms involved in the endothelium-dependent regulation of these processes are complex and involve hundreds of mediators, as reviewed elsewhere.^4−7^ Their concerted harmonized action featuring a healthy endothelium is essential for undisturbed functioning of the cardiovascular system, while endothelial dysfunction (ED) is recognized as a hallmark of various cardiovascular diseases. ED is closely interconnected with oxidative stress, decreased ·NO bioavailability, and vascular inflammation, representing unifying concepts for the underlying pathophysiology of cardiovascular morbidity and mortality.^8^ In fact, impaired ·NO signaling, vascular inflammation, and oxidative stress are key players in the pathogenesis of ED in various diseases.

Various classical risk factors (*e.g.*, hypercholesterolemia, hypertension, chronic smoking, or diabetes mellitus) or nonclassical risk factors such as the influence of the environment (*e.g.*, traffic noise exposure, ambient air pollution, and mental stress) or chronic inflammatory disease (*e.g.*, rheumatoid arthritis or psoriasis) can induce ED. Moreover, a few risk factors produce a synergistic effect on endothelial function as well as the associated cardiovascular prognosis.^8^ There is no doubt today that endothelial phenotype represents a real barometer of cardiovascular risk.^9^ However, despite over 5 decades of research, there is still a huge bench-to-bedside gap in endothelial biomedicine.^10^ One of the limitations on our understanding of endothelial physiology and pathophysiology is the huge complexity and heterogeneity of the endothelium and the paucity of experimental imaging methods that can be used for in-depth characterization of biochemical alterations of the endothelial phenotype.

Given the unique features of Raman spectroscopy to identify the chemical signatures of substances, here we critically review the ability of Raman microscopy to define the biochemical phenotype of ED. First, a summary of recent studies characterizing ED with the use of Raman spectroscopy is provided. Next, a critical review of the possibility of detecting subcellular alterations of the nucleus, mitochondria, endoplasmic reticulum, and lysosomes by a novel approach that combines Raman imaging with molecular reporters is described. Finally, we comment on imaging techniques based on both conventional spontaneous Raman scattering and the emerging technique called stimulated Raman scattering.

## 2 Raman Spectroscopic Markers of Endothelial Dysfunction Studied *Ex Vivo* in Isolated Vessels

Raman spectroscopy is based on the inelastic scattering of photons known as the Raman effect. It was first discovered by C. V. Raman in 1928.^11^ Raman scattering is induced by monochromatic light directed into the sample where photons interact with the molecules, which then emit photons of the same (Rayleigh), lower (Stokes), and higher energy (anti-Stokes) than the absorbed photons. The Raman spectrum is generated upon detection of the scattered photons in an inelastic way by a molecule. Each molecule has a unique spectrum in which certain bands correspond to specific functional groups and the intensity of the bands can be correlated with the concentration of the analyzed compound in a given sample. Therefore, Raman spectroscopy can be used both for qualitative and quantitative analyses.^12,13^

A set of relatively new techniques based on Raman spectroscopy has been developed to enhance the acquired Raman signal or to increase the sensitivity and/or selectivity of detection, especially in biological samples. An example of such techniques is resonance Raman (rR) spectroscopy, in which the laser excitation line is aligned to match the electronic transition of a targeted molecule. As a consequence, an enhancement of the intensity of the scattered Raman radiation by a factor of up to 10^6^ can be observed, improving the detection of a selected compound.^14^ Another technique that allows Raman signal enhancement of up to 10^8^ times and high imaging speed is stimulated Raman scattering (SRS) microscopy.^15,16^ SRS is a background-free nonlinear technique that requires the use of two ultrafast pulsed laser lines, denoted as the pump and probe. This technique has gained popularity because of its potential to improve sensitivity and reduce measurement time. Furthermore, electronic preresonance stimulated Raman scattering (epr-SRS) microscopy has been developed as a novel technique that benefits from the synergetic effect of rR and SRS.^17^ This technique holds great potential to utilize Raman reporters to increase remarkably the sensitivity of the Raman measurements.

Raman spectra of cells are complex since they contain information about all of the molecules present in the sample, but they are especially useful in studying lipids, proteins, and nucleic acids, providing insight into chemical structure and changes in the cells. Confocal Raman microscopy has many advantages over other widely used imaging methods such as fluorescence imaging; it has high chemical specificity and generally does not require labels that could influence the outcome of the study. Moreover, Raman spectroscopy allows imaging of cells in aqueous environments since water has a weak Raman signal.^11^ This makes Raman microscopy a reliable tool to study biochemical processes in cells and tissues, *e.g.*, to follow the development of a disease. Indeed, Raman spectroscopy has been successfully used in our previous studies to investigate the changes associated with ED using *in vitro* and *ex vivo* models of atherosclerosis, diabetes, and hypertension, often combined with complementary techniques.^18^

**Figure 1:**
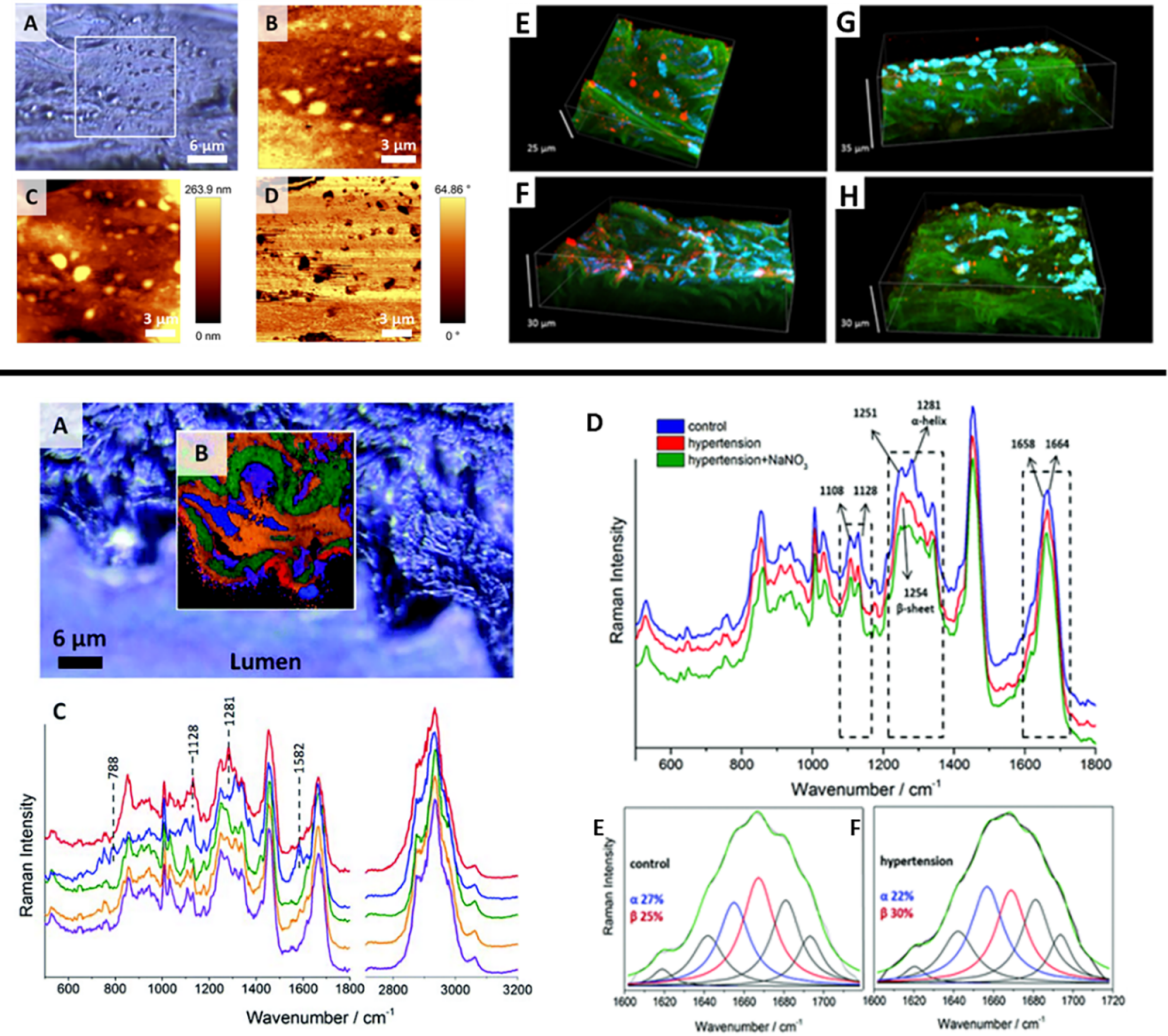
Raman imaging of endothelial dysfunction (ED) studied *ex vivo*. (top) Imaging of lipid rafts in *en face* aorta in db/db mice: (A) microphotograph of a studied tissue; (B) Raman image obtained by integration in the region 2830–3030 cm^–1^; (C) topography and (D) phase AFM images; (E–H) confocal micrographs of (E, F) db/db and (G, H) db+ tissue fragments showing endothelial caveolin-1 (red), cell nuclei (blue), and elastin (green). From ref (21). CC BY 4.0. (bottom) Raman investigation of a NO-deficient hypertensive murine model: (A) confocal micrographs of thoracic aorta cross-section of a control mouse; (B) cluster image and (C) average spectra of classes corresponding to the endothelium (red), cell nuclei (blue), and elastin fibers (green), along with (D) average spectra taken from the endothelium region of control (blue), hypertensive (red), and nitrate-treated (green) mice; (E, F) deconvolution of the amide I band for (E) control and (F) hypertensive mice, where the blue and red lines correspond to the α-helix (1656 cm^–1^) and the β-sheet (1668 cm^–1^), respectively, and the green line is the sum of all bands.^25^ Adapted with permission from ref (25). Copyright 2014 Royal Society of Chemistry.

For example, 3D confocal Raman imaging combined with atomic force microscopy (AFM) provides a valuable insight into biochemical content in relation to nanomechanics of ED. Through processing of the acquired Raman spectra, this technique offers valuable information on the chemical structures of the samples and the distribution of certain biological molecules (*i.e.*, lipids, proteins, *etc.*) while AFM offers insight into the samples’ nanomechanical properties, allowing the acquisition of comprehensive information on ED phenotype. Although the mechanisms of diabetes-induced ED have been addressed in multiple studies,^19^ 3D confocal Raman imaging combined with AFM of split-open (*en face*) aorta of a genetically modified murine model of type 2 diabetes mellitus (db/db) provided a novel insight into the chemical content and nanomechanical aspects of the endothelium.^20^ A significant increase in the area occupied by lipid rafts was observed, along with an overall increase in lipid content in the aorta of diabetic mice associated with an increase in the lipid to protein ratio (Figure 1, top).^21^ Interestingly, through Raman spectroscopy-based detection of protein and lipid bands in a cross section and *en face* aorta it was possible to distinguish between pathology and control.^22^ In fact, hierarchical cluster analysis (HCA) classified endothelial dysfunction in diabetes with 88% sensitivity and >94% specificity.

ED in hypertension is also linked to NO deficiency,^23,24^ similarly as in diabetes. However, label-free Raman imaging detected distinct alterations in the hypertensive state versus control as compared with diabetes.^25^ A significant change in the Raman spectra in the 1200–1400 cm^–1^ region due to alteration of the secondary structure of proteins in NO-deficient hypertensive mice was observed, where an increase in the intensity of the band at 1254 cm^–1^ and a significant decrease in the intensity of the band at 1281 cm^–1^ signaled an increase in the relative amount of the β-sheet structure relative to the α-helix structure (Figure 1, bottom). Furthermore, a decrease in the lipid to protein ratio upon pathology development was detected. While a nitrate supplement restored the balance in the lipid to protein ratio, the structural changes of endothelial proteins were not modified.^25^ HCA results enabled the discrimination between NO-deficient and control animal samples on the basis of the position and shape of the amide III band (1222–1374 cm^–1^) and the lipid to protein ratio, with both sensitivity and specificity of ca. 93%.^22^

In turn, in a murine model of atherosclerosis in apolipoprotein E and low-density lipoprotein receptor (LDLR) double-knockout (ApoE/LDLR–/−) mice,^26^ Raman-based features of ED were dominated by lipid signals. A significant increase in the intensity of the C–H stretching band (2800–3030 cm^–1^) by +17% was observed in the ApoE/LDLR–/–, mice indicating an increase in the intracellular lipid content. Moreover, compared with wild-type mice, endothelial cells from ApoE/LDLR–/– mice showed a significant increase in cortical stiffness, suggesting that impairment of NO-dependent function was linked to increased lipid endothelial accumulation and cortical stiffness.^27^

Raman spectroscopy also revealed that the tyrosine (Tyr) to phenylalanine (Phe) ratio was changed in ED in atherosclerosis.^28^ Tetrahydrobiopterin (BH4) is a key regulator of endothelial nitric oxide synthase (eNOS), and therefore, limitation of BH4 availability triggers ED and is reflected by changes in the synthesis of Tyr from Phe that depend on the presence of BH4 as a cofactor of phenylalanine hydroxylase (PAH).^29^ Accordingly, alterations in the Tyr to Phe ratio may indicate ED. The relative intensities of characteristic Raman bands of Phe (1004 cm^–1^) and Tyr (854 cm^–1^) were analyzed in samples taken from the aorta of atherosclerotic (ApoE/LDLR–/−) mice compared with 1-methylnicotinamide (MNA)-treated and control mice.^30^ The results showed that the Tyr to Phe ratio was significantly lower for ApoE/LDLR–/– samples compared with the control.^30^ Moreover, this ratio slightly increased in mice treated with MNA,^30^ an agent known to improve endothelial function in ApoE/LDLR–/– mice.^31,32^

Overall, Raman spectroscopy has the potential to detect spectroscopic markers of ED and to uncover biochemical changes associated with ED that to some degree seems to be disease-specific. Indeed, in our previous studies in animal models of ED in diabetes, hypertension, and atherosclerosis, markers of ED were specific for each of the studied diseases.^21,22,25,27^ Still, the subcellular origin of these changes could not be detected, and therefore, it was not possible to establish which organelle was involved in the development of ED in these models.

## 3 Label-Free and Labeled Raman Imaging of Cellular Organelles

One of the advantages of Raman microscopy is the ability to obtain information about the chemical composition of biological samples without the need to introduce dyes (labels). Label-free Raman imaging offers reliable information on the distribution and chemical composition of various biological components (*i.e.*, nucleic acids, proteins, lipids, *etc.*). However, their characteristic Raman bands can overlap. Therefore, even though this technique is capable of detecting several changes associated with the development of endothelial pathology, the sensitivity and selectivity of subcellular imaging is limited and could be significantly improved using molecular Raman reporters, a method called labeled Raman imaging. Such reporters would ideally have a targeting moiety specific to certain organelles and a Raman reporting moiety that would enhance the obtained Raman signal or give a strong band in the biologically silent spectral region (1800–2800 cm^–1^). Alternatively, isotopic labeling of certain molecules (*e.g.*, substitution of protons with deuterium) has the potential to detect cellular responses under various conditions since molecular vibrations of isotope-labeled groups are different than all of the others. It is worth mentioning that using molecular Raman reporters requires an extra step of sample treatment, during which the reporters’ properties such as their light sensitivity, solubility, cellular uptake, and possible cellular effects should be taken into consideration, although ideally Raman reporters are desired to be inert or have a negligible effect on the samples.

The possibilities and limitations of imaging the interior of cells with label-free or labeled approaches are discussed below. The cell organelles (*i.e.*, mitochondria, endoplasmic reticulum, nucleus, and lysosomes) were selected on the basis of the available literature indicating the applicability of both approaches as well as their importance in the context of mechanisms of ED.^33−35^Table 1 presents selected compounds and indicates their molecular structures responsible for the Raman signal and their affinities to specific subcellular organelles separately. The structures of commercially available dyes utilized in EPR-SRS are not shown.

**Table 1:**
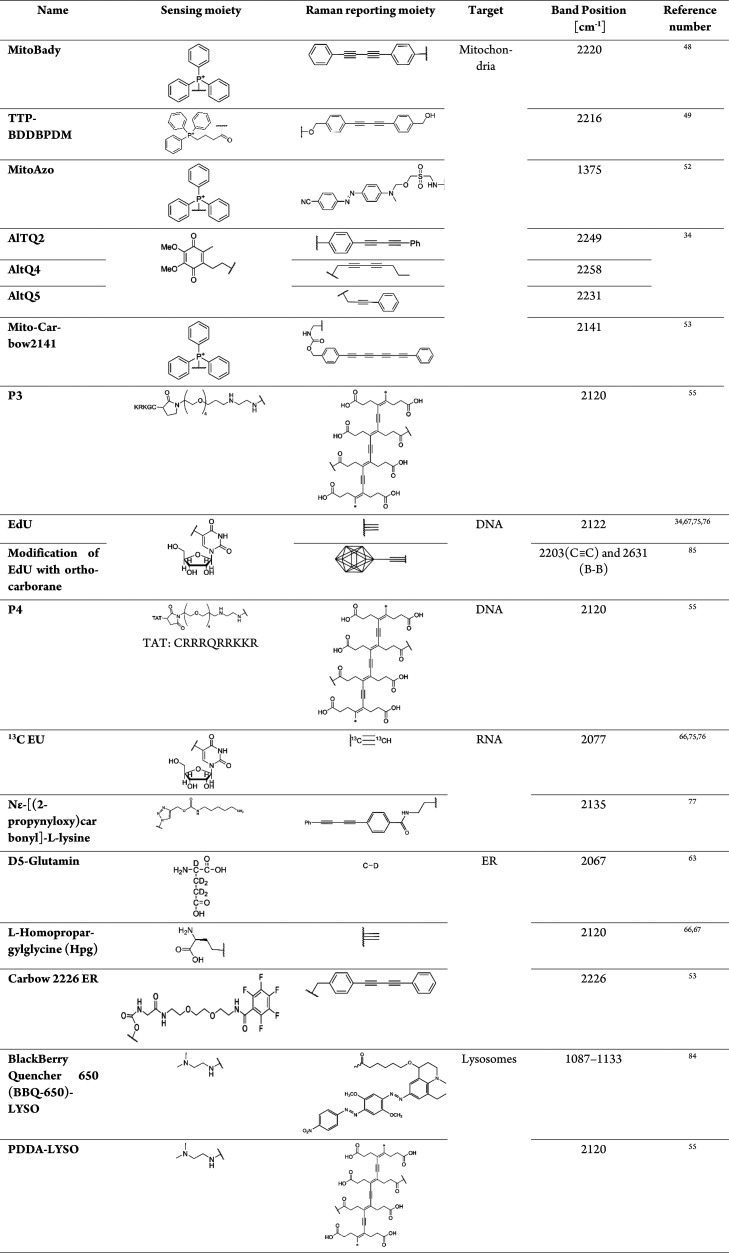
Summary of Raman Reporters

### 3.1 Mitochondria

Endothelial cells undergo mainly anaerobic glycolytic metabolism, but the role of mitochondrial oxidative phosphorylation to produce ATP and regulate the endothelial phenotype is increasingly appreciated.^36−38^ In the context of ED, mitochondrial reactive oxygen species (ROS) are fundamental in the regulation of vascular signaling, such as endothelial proliferation, shear-stress-induced vasodilation, and apoptosis. On the other hand, an excess of ROS has multiple harmful effects on mitochondrial DNA, on the bioavailability of NO, which reacts with superoxide to form peroxynitrite, and on lipid peroxidation. Increased mitochondrial ROS are also implicated in cell death pathways like programmed necrosis (necroptosis) and apoptosis. There are a number of pathophysiological situations whereby alterations of mitochondrial function contribute to ED, such as hyperglycemia and diabetes,^36,39−41^ Ang-II-induced ED,^42−44^ and atherosclerosis.^36^

The presence of cytochrome complex inside mitochondria allows for selective label-free Raman imaging of its distribution based on a set of characteristic bands (at approximately 750, 1130, 1310, and 1590 cm^–1^), especially when the measurements are performed under rR conditions resulting in enhancement of the cytochrome *c* bands.^45,46^ Apoptosis occurring in the mitochondrial pathway is characterized by the release of cytochrome *c* to the cytoplasm, which shows the utility of Raman spectroscopy studies of cytochrome distribution.^45^ However, under off-resonance conditions (using excitation light of wavelength above 550 nm) characteristic cytochrome *c* bands may be masked by Raman signals from dominant intracellular components (*e.g.*, proteins and lipids).^47^ Therefore, we emphasize that label-free Raman imaging of cytochrome is limited to measurements carried out under resonance conditions (laser line in the range of the cytochrome *c* absorption band) and for living cells. Since mitochondria occupy only 2–6% of the endothelial cytoplasm volume, nonresonance imaging may result in an inability to detect the cytochrome *c* bands.^37,47^

**Figure 2:**
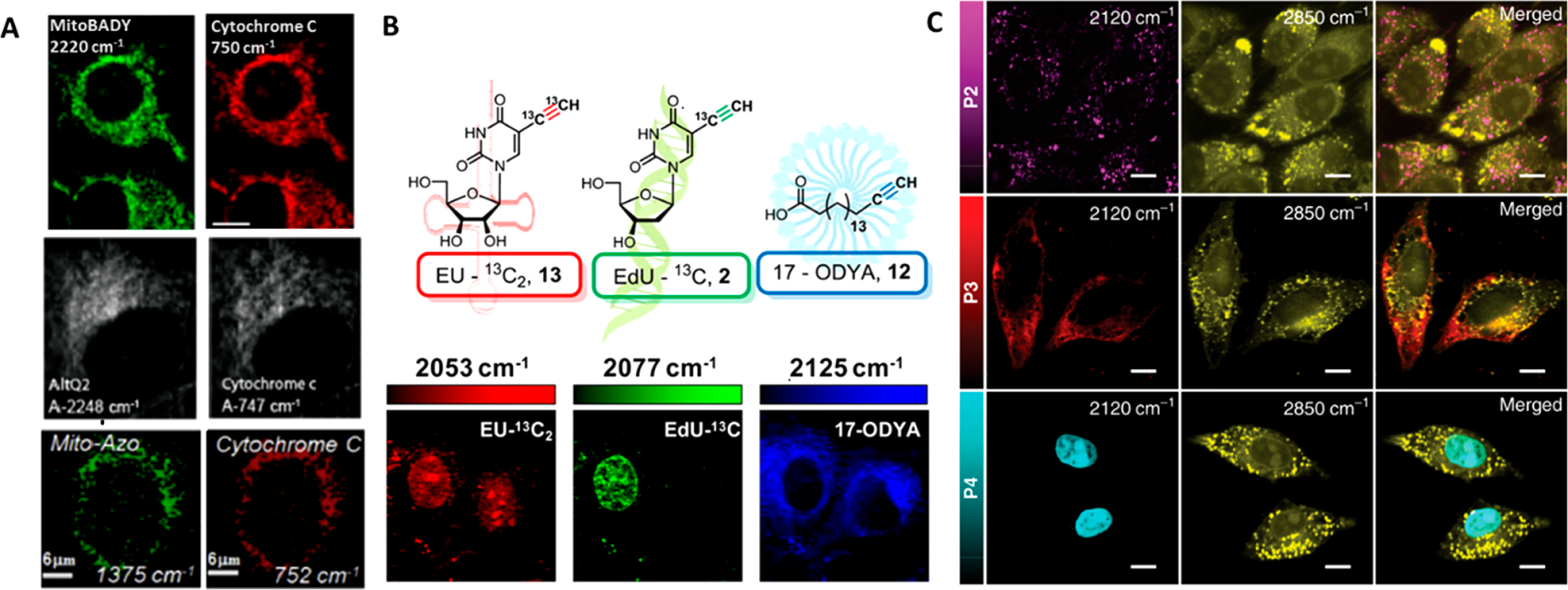
Raman subcellular imaging using molecular reporters. (A) Raman imaging of mitochondria probes MitoBADY, AlTQ2 and Mito-Azo (left) and cytochrome *c* (right) in live Hela cells. Adapted with permission from refs (48), (34), and (52). Copyright 2014 Elsevier, 2012 American Chemical Society, and 2015 Elsevier, respectively. (B) Structures of the isotopically edited RNA probe EU-^13^C_2_ (red), DNA probe EdU-^13^C (green), and fatty acid probe 17-ODYA (blue) and SRS imaging of RNA, DNA, and fatty acyl derivatives in live HeLa cells by spectral targeting of these isotopically edited alkyne reporters. Adapted from ref (76). Copyright 2014 American Chemical Society. (C) SRS images of HeLa cells obtained using lysosome-, mitochondria-, and nuclei-targeting PDDA (denoted as P2, P3, and P4, respectively) at 2120 cm^–1^, which corresponds to the alkyne band maximum, and overlay with the lipid distribution (2850 cm^–1^). Scale bars: 10 μm. From ref (55), CC BY 4.0.

Therefore, the application of so-called Raman reporters (also known as Raman probes or Raman tags) that give a strong Raman scattering peak in the spectrally silent region (1800–2800 cm^–1^) enables more specific Raman imaging of subcellular structures. Spontaneous Raman and SRS studies showed the potential of bis(aryl)butadiyne (BADY) and its derivatives combined with triphenylphosphonium cation (TPP^+^) as a mitochondrial targeting moiety (Figure 2A).^48,49^ The charge brought by TPP^+^ in MitoBADY is easily targeted to mitochondria because of highly negative mitochondrial transmembrane potential, as proved by its colocalization with a signal characteristic for cytochrome *c* and MitoTracker Green fluorescence dye.^48,50^ SRS studies of the diyne molecule TTP-BDDBPDM in living HeLa cells reported that maximum accumulation within cell decreased by 37% after addition of the mitochondrial membrane potential dissipating agent CCCP.^49^ Moreover, TPP^+^ structures can be found in combination with radical scavengers, imaging agents, or a superoxide probe as examples of compounds with various functions.^50,51^ Prasad’s group applied a similar approach that coupled the above-described TPP^+^ with the azobenzene-based probe Am-CN-Azo-OH, which allowed enhancement of the 1375 cm^–1^ band of MitoAzo due to electronic resonance of Am-CN-Azo-OH under excitation with 532 nm light (Figure 2A).^52^ To study mitochondria distribution, analogues of coenzyme Q, an important compound in the electron transport chain in mitochondria, have been used. AlTQs were designed to have similar calculated logP (clogP, where logP stands for the partition coefficient of a molecule between an aqueous phase and a lipophilic phase) and contain active groups that give Raman bands in the silent spectral region (Figure 2A).^34^

Multiplex imaging, defined as simultaneous detection of multiple species with high sensitivity, requires narrow, well-resolved bands. Polyyne-based reporters (denoted as Carbow) overcame the problem of possible overlapping with bands originating from other cellular components that have been observed in the case of EPR-SRS imaging with commercially available dyes. By modification of isotopes and the number of triple bonds, Carbows cover a spectral range of 2226–2066 cm^–1^. Functionalization of 4-yne (Carbow2141) with TPP^+^ succeeded in visualization of mitochondria distribution.^53^ Multiple triple bonds in the polyyne structure influence the signal intensity and enhance the chemical contrast. Water-soluble poly(deca-4,6-diyneoic acid) (PDDA) functionalized with organelle-targeting groups showed the potential to image subcellular compartments with lower laser power. In order to target the mitochondria, the PDDA was conjugated with CGKRK, a tumor-specific homing peptide that has been shown to bind to mitochondria of tumor cells and tumor vessel endothelial cells.^54^ Colocalization of the 2120 cm^–1^ Raman signal with a fluorescence image confirmed mitochondrial targeting (Figure 2 C).^55^

Detection under electronic preresonance conditions is conducted with careful laser frequency tuning into the region of electronic preresonance, which was shown for several near-infrared-absorbing commercially available fluorescent probes. Mitochondria distribution was imaged with MitroTracker Deep Red, ATTO740 immuno-labeled protein Tom20, and rhodamine 800. The intensities of the respective bands at 1604, 1642, and 1652 cm^–1^ were elevated, allowing imaging of the intracellular distribution of these dyes.^56^

The multiplicity of reported approaches to develop suitable mitochondrial targeting and reporting moieties fulfilling the criteria of nontoxicity, bioavailability, and sensitivity underline the importance of mitochondrial-targeted mechanisms and active research on this topic. Interestingly, the mechanism underlying preferential accumulation of the majority of the above-presented probes utilizes the affinity of the negatively charged mitochondrial membrane to cationic targeting moieties. However, their influence on mitochondrial functions must be considered membrane potential and mitochondrial function must be considered. Another approach worth considering is the application of reporters such as AlTQs that mimic the structure of important mitochondrial mechanisms or utilize functionalized peptides.^34,55^

### 3.2 Endoplasmic Reticulum

The endoplasmic reticulum (ER) is the largest organelle in the cell. In particular, the rough ER is involved in the synthesis, folding, and post-translational modification of proteins.^3^ Altered function of the ER, so-called ER stress, has been repeatedly linked to the development of ED.^2,3^ An example of ER stress activation is faced when the unfolded protein response (UPR) fails to maintain the balance between the load of new proteins to be folded and the folding capacity of the ER or when the UPR is chronically activated. The UPR activates pathways to reduce the newly synthesized protein load, enhance the ER folding capacity, and activate the ER-associated degradation machinery to dispose of irremediably misfolded proteins.^2,3^ All of the pathways induce the transcription of proinflammatory signals activating the proinflammatory regulator nuclear factor κB (NF-κB). Each pathway acts against the ER stress but at the same time interacts with ROS, cell death pathways, and inflammatory signaling that are involved in the development of ED. An example of the relation between ER stress and ED can be found in the state of hyperglycemia, where glucose-induced expression of inflammatory cytokines is accompanied by UPR activation and is mitigated by chemical chaperones (*e.g.*, phenylbutyric acid (PBA)).^2,57^ To summarize, the involvement of the ER in ED comprises two main steps. First, ER stress is induced through persistent activation of the UPR by numerous substances. Free fatty acids (*e.g.*, long-chain saturated fatty acids), LDL oxidation (oxLDL) and oxysterols, flow disturbance and homocysteine, and angiotensin II lead to activation of the UPR with different mechanisms.^58^ Second, the ER stress state leads to ED, impairing the balance in the vasoactive mediators (*e.g.*, leading to hypertension), triggering inflammatory responses (*e.g.*, activation of transcription factor NF-κB), and increasing ROS production that promotes oxidative stress (*e.g.*, as involved in the atherogenic sequence) and acts as a positive closed loop.^2,3^ Long-lasting problems with balance maintenance of this process cause so-called ER stress and may lead to cell death.^59^

Many pro-apoptotic and anticancer drugs induce endothelial ER stress. One of them is tunicamycin (TU), which was reported to induce endothelial toxicity and vascular dysfunctions.^60^ TU treatment of human aortic endothelial cells (HAoEC) showed an increase in the Raman bands characteristic of proteins at 985 cm^–1^ (tryptophan), 1235 cm^–1^ (amide III), and 1342 cm^–1^, while a decrease in characteristic phospholipid bands was observed at 715 cm^–1^ (choline N^+^(CH_3_)_3_) and 1072 cm^–1^ (PO_4_^3–^). Additionally, this result was confirmed by a decrease in the intensity of lipid bands at 2855 and 2894 cm^–1^. Surprisingly, Raman imaging allowed the observation of increased volume of the ER under treatment with TU, which may indicate abnormal protein and TU storage. A slight but not statistically significant increase in phospholipid content was observed during the early state of apoptosis under incubation with the apoptotic factors Fas ligand (FasL) and cycloheximide (CHX) in a semiquantitative analysis of the whole cell. More detailed analysis of each cellular compartment confirmed a decrease in phenylalanine content (1007 cm^–1^). In contrast to CHX, FasL-treated cells in the ER area exhibit a red shift of the amide III band to 1254 cm^–1^, indicating changes in protein secondary structure, which might correspond to caspase activity. However, the amide III signal maximum at 1266 cm^–1^ after CHX treatment suggests a different mechanism of apoptosis induction by CHX.^61^

SRS has been used to visualize the synthesis of proteins *de novo* in the ER and their incorporation^62^ as well as already existing protein degradation by incubation of cells in optimized medium containing single or multiple stable isotope-labeled amino acids (SILACs).^63,64^ Protein degradation can be observed either in the fingerprint by introducing ^13^C-phenylalanine with the ring-breathing band at 968 cm^–1^^64^ or in the cell-silent region using a mixture of deuterated leucine, isoleucine, and valine or arginine, lysine, and methionine.^63^ This approach was also successfully applied to visualize protein biosynthesis in HeLa cells transinfected with plasmid coding a Huntington probe with a fluorescent label. A glutamin-*d*_5_-containing medium was recently used by Miao and Wei to image mutant Huntington protein aggregates with a long polyglutamine chain at 2067 cm^–1^.^65^ Besides SILACs, the alkyne-tagged analogue l-homopropargylglycine (Hpg) can be used to image newly synthesized proteomes at 2120 cm^–1^.^66,67^ Biosynthesis of proteins and other cellular components like lipids can be studied with D_2_O, as confirmed with Raman spectroscopy for bacteria^68,69^ and with SRS in *in vitro* and *in vivo* murine models.^70^ Moreover, SRS has been used to characterize the ER membrane and prove that metabolites of external saturated fatty acid induce the formation of domains with solid characteristics, which is impossible with a bulky fluorescent probe such as BODIPY.^71^

It is noteworthy that the literature describing imaging of only the ER with Raman reporters is rather scarce. ER imaging with SRS was reported using pentafluorobenzamine as the targeting moiety coupled with 4-yne.^53^ To date, one Raman probe has been described that uses glibenclamide, a popular medicine for diabetes mellitus 2 that binds to the sulfonylurea receptors of ATP-sensitive K^+^ channels, mainly on the ER.^51^ The shortage of targeting moieties for direct ER imaging emphasizes the challenge that both Raman and fluorescence spectroscopy are facing in the field of ER studies and underscores the necessity of developing alternative probes that allow direct imaging of the ER.

### 3.3 Nucleus

Endothelial inflammation is associated with the increased expression of various pro-inflammatory cytokines and pro-thrombotic molecules and activation of various transcription factors such as NF-κB involving activation of nuclear transcription and chromatin rearrangement.^8^ On one side, it promotes the pro-inflammatory phenotype, and on the other side, it leads to apoptosis. Pathways leading to repair of DNA damage are then activated (DNA damage response (DDR)) that recruit specific DNA repair factors and effectors (*e.g.*, poly[ADP-ribose] polymerases (PARPs)) to repair DNA or to induce senescence and apoptosis. However, compression of chromatin suppresses expression of some DDR proteins and is possibly linked with vascular calcification.^72^

Raman spectroscopy has been used to investigate the changes in the nuclei and nucleoli by identifying the bands corresponding to nucleic acids that arise from nucleotide and sugar–phosphate backbone vibrations, specifically the bands at ca. 788 cm^–1^ (phosphodiester bonds in DNA), 813 cm^–1^ (phosphodiester bonds in RNA), and 1095 cm^–1^ (phosphodioxy group, PO_2_^–^).^73^ In another study, endothelial cells were stimulated with FasL and CHX.^61^ It has been shown that during early apoptosis a significant decrease in the protein content is observed. However, in the FasL-stimulated cells, a significant increase in the amounts of nucleic acids was noticed (a marker band at 785 cm^–1^) that was due to the increase in chromatin condensation.^61^

One of the commercially available Raman reporters that is targeted to the nucleus is the alkyne-tagged thymidine analogue 5-ethynyl-2′-deoxyuridine (EdU). EdU is widely used for copper(I)-catalyzed azide–alkyne [3 + 2] cycloadditions with a fluorescent probe to study cell proliferation. It easily penetrates cells or tissue samples and is incorporated into double-stranded DNA.^67,74^ Alternatively, EdU has an intense alkyne peak at 2122 cm^–1^.^34,66,67,75,76^ The concentration required to successfully image incorporation of EdU is at the same level as recommended by commercial EdU-based fluorescence labeling kits (*i.e.*, 20 μM) for HeLa cells incubated with EdU for 3 or 21 h.^75^ It is worth underlining that EdU is incorporated into newly synthesized DNA, as evidenced by the lack of the 2122 cm^–1^ band for cells incubated with EdU in the presence of hydroxyurea, an inhibitor of DNA synthesis.^66,67^ Modification with ^13^C isotopic substitution of the alkyne (^13^C≡^13^C EdU or ^13^C EU) in the RNA precursor and the alkyne-labeled lipid 17-octadecynoic acid allowed for simultaneous detection of tags at 2015, 2077, and 2125 cm^–1^ (Figure 2B).^76^ However, the conventional application of EdU as a click reaction substrate to react with a commercially available near-infrared absorbing dye such as Cy5.5 or ATTO 740 was an interesting approach using EPR-SRS. The DNA distribution was demonstrated using bands at 1642 and 1626 cm^–1^, respectively.^56^ Additionally, an important nuclear protein, histone H2B, was imaged using EPR-SRS with appropriate labeling using far-red silicon-rhodamine displaying a strong band at 1610 cm^–1^.^56^ Except for EdU, which shows the distribution of newly synthesized DNA, conventional SRS nucleus imaging is also possible with PDDA conjugated with the cationic transactivator of transcription (TAT) peptide CRRRQRRKKR (Figure 2C).^55^ Modified molecules often do not undergo biochemical processes in cells, which may lead to toxicity or prevent the study of more sophisticated biological functions. However, the unnatural amino acid (UAA) *N*ε-[(2- propynyloxy)carbonyl]-l-lysine was incorporated into histone 3.3–EGFP protein, expression of which in the nucleus was visualized at 2135 cm^–1^, showing the potential of genetically targeted proteins in organelle imaging.^77^ Although label-free Raman microscopy enables the visualization of cell nuclei, it does not distinguish between the nucleic acids since the detection is mainly based on the peak at ca. 785 cm^–1^ that corresponds to the ring-breathing modes of both DNA and RNA. This point could be overcome using two different probes for DNA and RNA detection (EdU and EU, respectively). Nonetheless, the development of new Raman reporters that allow faster and better imaging of DNA and RNA simultaneously would be of great interest.

### 3.4 Lysosomes

Lysosomes are involved in the complex cellular machinery of autophagy. The basal level of autophagy occurs constantly in endothelial cells with the primary function of clearance of misfolded proteins and dysfunctional organelles that can be harmful to the cell (*e.g.*, damaged mitochondria producing excessive ROS). Moreover, autophagy is elicited by different stimuli coming from both the intra- and intercellular environments. These stimuli span from metabolic and redox stressors, as in the case of nutritional starvation, to encompass also dysregulation of ROS, hypoxia, DNA damage, and mechanical shear stress. Under such circumstances, the role of autophagy is generally cytoprotective through the activation of pathways aimed to preserve the physiological balance. These different functions cover a range from dynamic adjustment of the bioenergetic and biosynthetic needs of endothelial cells when metabolic stress, nutritional starvation, or angiogenesis occur to playing a role in the production of eNOS and the secretion of von Willebrand factor from Weibel–Palade bodies in order to preserve the homeostasis balance.^78−80^ However, dysregulated autophagy is also involved in ED.^80^ Even though the mechanisms involved are not completely understood, depending on the circumstances autophagy can switch from cytoprotective function to those promoting endothelial dysfunction. Given the importance of autophagy in ED, it is worth remarking that even if different types of autophagy do exist (chaperone-mediated, macro-, and microautophagy), they are all related to lysosomes.^81−83^

Because there is no Raman marker for lysosomes, detection of alterations at the lysosomal level in a label-free manner is not possible. Recently, resonance Raman probes based on BlackBerry Quencher 650 (BBQ-650) conjugated with *N*,*N*-dimethylethylenediamine as a lysosome targeting moiety to visualize this cellular compartment were suggested. Application of the 633 nm laser line with energy close to the absorption maximum at 650 nm caused an increase in the intensity of the bands.^84^ The same idea was applied to direct PDDA and 2-yne to lysosomes (Figure 2C).^55^ Because of elevated lysosomal accumulation of methylene blue, it was also suggested as a promising lysosomal Raman reporter that can be imaged with EPR-SRS tuned to the band at 1630 cm^–1^.^56^ The mechanism underlying preferential lysosomal accumulation of probes is protonation of the weak base used as the targeting group.^84^ Membrane-impermeable protonated amines are selectively trapped in the lysosomes. As in the case of most of the mitochondria-targeting moieties, excessive changes in the lysosome environment and alkalization may alter their functions in long-term tracing. Nevertheless, further optimization of the structure–function relationship of reporters is needed for such important organelles to be used more widely for imaging.

## 4 Detection of Cellular Uptake and Accumulation of Specific Labeled Molecules

**Figure 3:**
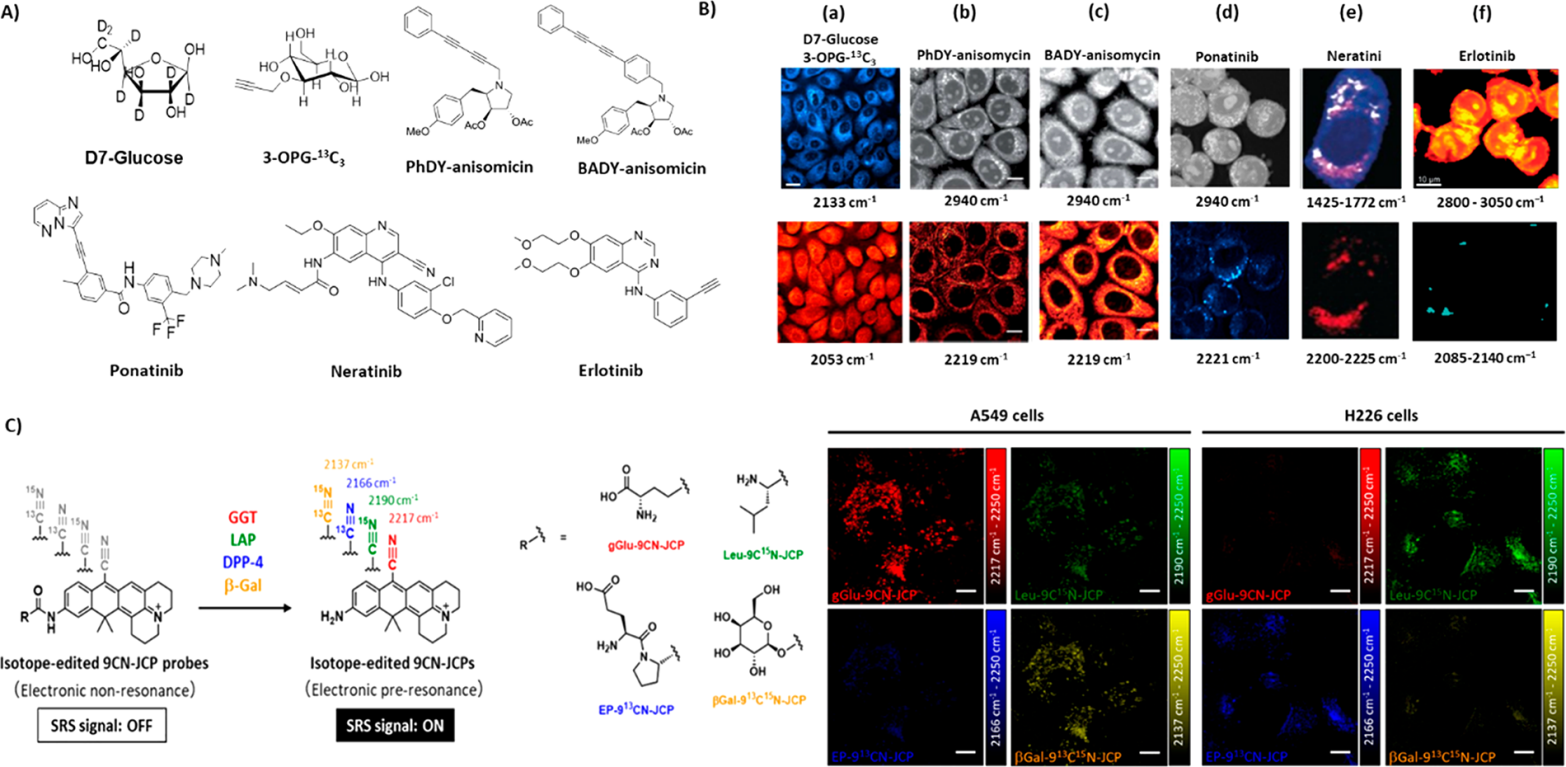
Compounds used to study intracellular transport tracking. (A) Chemical structures of D7-glucose, 3-OPG-^13^C_3_, BADY-anisomycin, PhDY-anisomycin, and ponatinib. (B) SRS imaging of (a) PC-3 cells treated with D7-glucose for 48 h and then with 3-OPG-^13^C_3_ for 2 h, indicating glucose incorporation (2133 cm^–1^, cyan hot), glucose uptake (2053 cm^–1^, red hot); (b) fixed SKBR3 cells treated with BADY-ANS (100 μM, 30 min); (c) PhDY-ANS (100 μM, 30 min); (d) KCL22 cells treated with ponatinib (5 μM, 1 h); (e) SK-BR-3 cells treated with neratinib (5 μM, 8 h); (f) SW480 cells treated with erlotinib (100 μM, 12 h). Images were acquired at 2940 cm^–1^ (CH_3_, proteins), 2219 cm^–1^ (C≡C, PhDY-/BADY-ANS), and 2221 cm^–1^ (C≡C, ponatinib). (C) Structures of the isotope-edited EPR-SRS probes 9CN-JCP (red), 9C^15^N-JCP (green), 9^13^CN-JCP (blue), and 9^13^C^15^N-JCP (yellow) and SRS images of probes incubated with the A549 and H226 cell lines acquired at 2217 cm^–1^ (gGlu-9CN-JCP, red), 2190 cm^–1^ (Leu 9C^15^N-JCP, green), 2166 cm^–1^ (EP-9^13^CN-JCP, blue), and 2137 cm^–1^ (β-Gal-9^13^C^15^N-JC, yellow). In panel (B), part (a) is adapted with permission from ref (86). Copyright 2017 Royal Society of Chemistry. Parts (b) and (c) are from ref (87). CC BY 3.0. Part (d) is adapted from ref (88). Copyright 2019 American Chemical Society. Part (e) is adapted from ref (90). CC BY-NC-ND 4.0. Part (f) is reproduced with permission from ref (91). Copyright 2013 Royal Society of Chemistry. Panel (C) is adapted from ref (89). Copyright 2020 American Chemical Society.

Undeniably, glucose is the major energy source for cells, and alterations in glucose metabolism lead to diabetes.^3^ It is worthy of note that increased cellular glucose uptake is used as diagnostic indicator of tumor cells under PET or NMR investigation. Thus, techniques allowing for cost-effective imaging of the uptake and development of new effective glucose-based probes or other probes to study the uptake of other bioenergetic substrates are very attractive. SRS was used to study the uptake of substituted D7-glucose based on the distinct bands at 2060 and 2250 cm^–1^ and the analogue 3-*O*-propargyl-d-glucose (3-OPG) at 2129 cm^–1^ using *in vitro* models as well as *ex vivo* measurements of the brain (Figure 3A,B(a)).^93^ In order to minimize the possibility of band overlap, 3-OPG was modified with ^13^C≡^13^C (denoted as 3-OPG-^13^C_3_), which changed the characteristic band position to 2052 cm^–1^, and was simultaneously imaged *in vitro*. Ratiometric images (C–D/^13^C≡^13^C) allowed comparison of the efficiency of glucose incorporation into biomass *in vitro* and classification of the anabolic activity of biomass synthesis in descending order for normal prostate, kidney, cancer prostate, and glioblastoma cell lines.^86^ Further studies showed that lipids, nucleic acids, and proteins isolated from cells incubated in D7-glucose contained deuterium in their structure, suggesting *de novo* synthesis of D7-glucose. Then, multichannel SRS imaging with a signal-unmixing method separated the signals corresponding to the synthesis of lipids, proteins, and glycogen *de novo* (denoted as CD_L_, CD_P_ CD_G_, respectively). The ratio of CD_P_ to CD_L_ indicated different glucose metabolic activity in cardiac muscle, fat tissue, and liver of mice fed with D7-glucose, which is consistent with their metabolic activity. A similar approach applied to melanoma cell lines with varying degrees of cellular differentiation revealed differences in glycogen-accumulation phenotype and metabolic activity.^94^ Additionally, deuterated fat accumulation was observed in the intestines of mice pup breast-fed by a mother drinking deuterated water.^95^ Beside glucose SRS, peracetylated *N*-(4-pentynoyl)mannosamine (Ac4ManNAl) glycan can be studied with SRS.^67^

Accumulation of lipids in lipid droplets (LDs) and altered composition and size of LDs have been recognized as important pathophysiological elements of ED.^18^ The repertoire of LDs identified in ED include LDs rich in highly unsaturated lipids, which are assigned to inflammation, as well as LDs featured by more saturated lipids linked to apoptosis, with increased content of cholesterol and phospholipids.^96^ However, the mechanisms of their accumulation and the mechanisms by which LDs contribute to ED are not clear. Tracking of D-38 cholesterol metabolism was investigated by using SRS, giving the advantage of high signal intensity over D-7 cholesterol. D-38 cholesterol was shown to have no effect on cellular processes and underlined the heterogeneity of composition of neutral lipids between single LDs. Moreover, it allowed for discrimination of the ratio of esterified to nonesterified cholesterol. Accumulation of cholesterol in LDs was recognized as a mechanism preventing toxicity and overaccumulation of cholesterol in membranes, which affects their stiffness.^97^ Another promising cholesterol-based SRS probe, tagged with a phenyl diene, was shown to undergo esterification and storage in LDs, proving that it mimics cholesterol behavior *in vitro* and *in vivo*.^98^ Other factors influencing changes in LD composition such as the presence of deuterated and alkylated fatty acids were widely studied by SRS, allowing the determination of fatty acid transport and a description of their role in the cell.^99−102^ Lipidic structures, including the ER and nuclear envelope, were visualized in endothelial cells under inflammation with astaxanthin, a carotenoid displaying resonantly enhanced bands at 1159 and 1523 cm^–1^ in spontaneous Raman spectroscopy using the 532 nm laser line.^103^ The phospholipid distribution, however, was studied using either deuterated (D9)^104^ or alkyne-tagged choline.^66^

The SRS technique was also reported to be promising to establish transport, accumulation, and mechanism of action not only of bioenergetic substrates and lipids but also small biologically active molecules.^87^ Anisomicyn activates the mitogen-activated protein kinase pathways JNK/SAPK1 and p38/SAPK2, causing inhibition of protein synthesis. Their modification with bis(aryl)butadiyne (BADY-ANS) or 5-phenyl-2,4-pentadiyne (PhDY-ANS) were observed at 2219 cm^–1^, showing the cytoplasmatic distribution (Figure 3A,B(b,c)). Additionally, the SRS signal is colocalized with the ER-Tracker fluorescent dye distribution, indicating that the labeled drugs might bind to ribosome subunits in a similar way as anisomycin.^87^ Other promising anticancer-antimycin-type depsipeptides labeled with PhDY were successfully imaged with SRS. Structures containing a macrocyclic nine-membered ring with an amide linkage to a 3-formamidosalicylate unit and a PhDY label were detected at 10 μM concentration, while 50 μM PhDY induced absolute intracellular concentrations as high as 1.74 mM. SRS imaging was in agreement with the ER-Tracker fluorescent dye distribution. This colocalization agrees with one of its known direct protein targets, Bcl_2_, an anti-apoptotic protein localized primarily in the ER. Interestingly, no significant colocalization with MitoTracker was observed, despite the known antimycin activity in mitochondria, though its strong correlation with lipids was presented.^105^ The authors of this study highlighted that modification of drugs with a Raman targeting group (here PhDY) and SRS imaging should be considered as a relevant method of drug detection.

Interestingly, some drugs have functional groups allowing for their direct detection in the silent region of the Raman spectrum without the need to add a Raman reporter moiety. This is the case with neratinib^90^ (Figure 3A,B(e)) and erlotinib^91^ (Figure 3A,B(f)), which are tyrosine kinase inhibitors (TKIs) that target epidermal growth factor receptor (EGFR) and are both detected by spontaneous Raman spectroscopy. SRS-based measurements of this type reduce the time required for intracellular drug detection compared with classical analytical methods (Figure 3A,B(d)). Furthermore, this approach could allow the drug intracellular distribution to be studied. Comparison with the LysoTracker and LysoSensor distribution confirmed the accumulation of the TKI within acidic structures of the cell.^88^ Visualization of drug accumulation can be performed using principally a fingerprint region of the spectrum collected from the stimulated cell. SRS studies indicated an increased accumulation of nilotinib and imatinib in lysosomes in contrast to other kinase inhibitors (*i.e.*, GNF2 and GNF5).^106^ In view of the evidence for the endothelial toxicity of some TKIs and many other anticancer agents,^107,108^ SRS-based studies on their uptake and subcellular distribution in the endothelium might perhaps offer a novel insight into their endothelial action and be useful in studies aimed to develop endothelium-safe chemotherapeutics.^107^

Other interesting applications of Raman probes have proved to be useful to determine enzyme activity *in vitro.* Indeed, enzyme activities in two human lung carcinoma models were determined using Raman probes. Enzymatic substrates such as γ-l-glutamyl(gGlu), l-leucyl (Leu), l-glutamyl-l-propyl (EP), and β-d-galactosyl (β-Gal) conjugated with the isotopically substituted nitrile group of the SRS-active scaffold 9CN-JCP upon reaction with the appropriate enzymes exhibited sharp Raman bands at 2217, 2190, 2166, and 2137 cm^–1^, respectively (Figure 3C). With probes at concentrations of 10–20 μM, the distribution and relative activity of four enzymes were imaged simultaneously using SRS. Overall, the detected enzyme activities were in agreement with their expression levels as assessed by PCR.^89^

**Figure 4:**
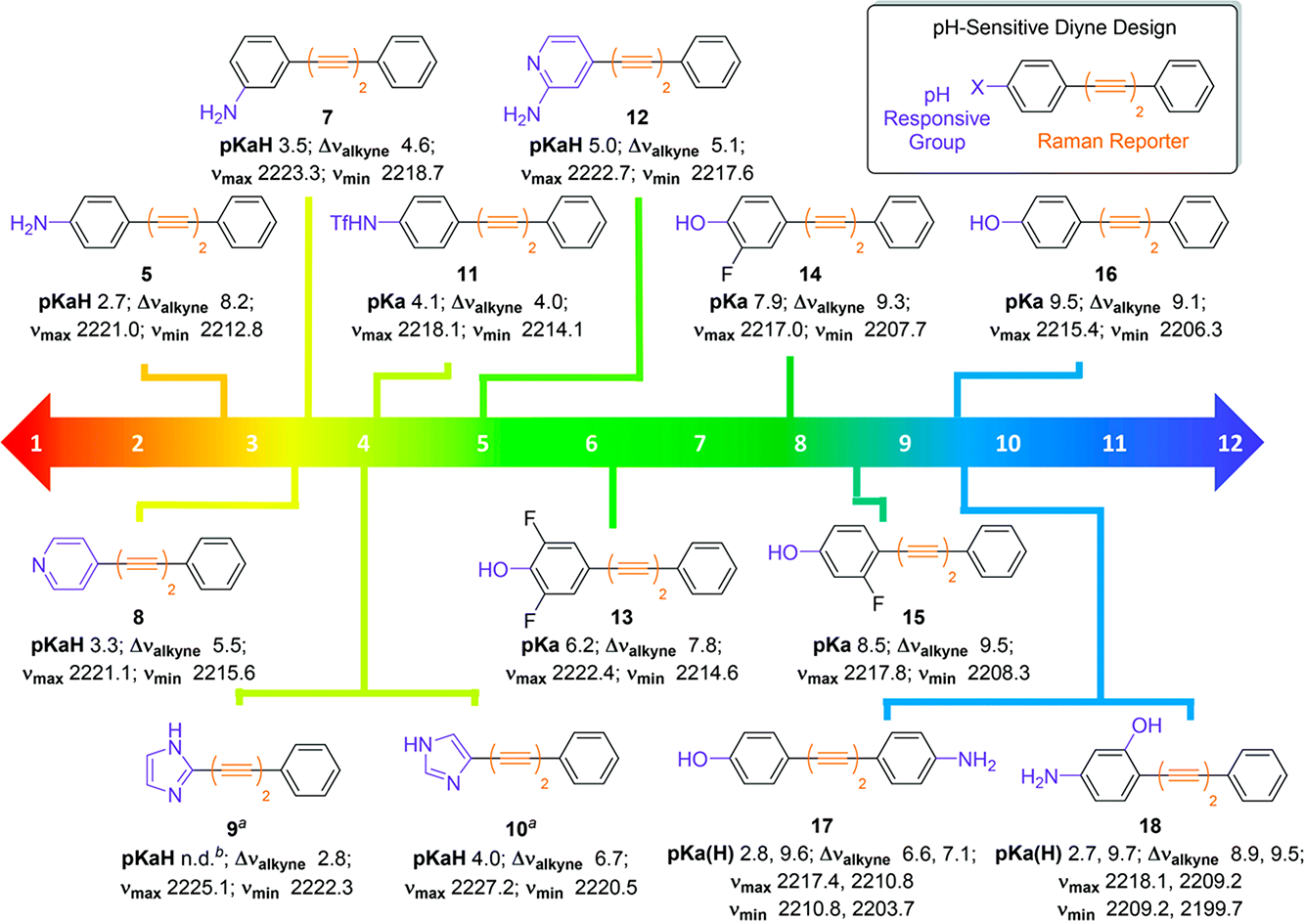
Structures of SRS ratiometric bis(aryl)butadiyne-based pH probes. Values of p*K*_a_(H) and the maximum and minimum band positions of the alkyne (ν_max_and ν_min_) and difference between these two values (Δν_alkyne_) are shown for each probe. Notes: ^*a*^experiments were carried out at a compound concentration of 200 μM; ^*b*^p*K*_a_(H) could not be determined. From ref (92). CC BY 3.0.

The application of Raman reporters also offers insight in detecting and monitoring the pH in the cell milieu. Regulation of intracellular and extracellular pH is essential for proper cell proliferation, protein synthesis, and metabolism. Thanks to their high Raman cross section, BADY-labeled compounds were found to be useful in multiplex-type measurements. The general idea of this approach is to combine a Raman reporting group (here BADY) with a pH-responsive group. The change in pH shifts the position of the band of the diyne group assigned to a specific pH value. As a result, 13 compounds were designed to cover the full pH range (Figure 4). The authors described the quantification of the intramitochondrial pH using the 13 compounds imaged inside human adenocarcinoma cells (PC3) in the presence of nigericin, a common fluorescence pH standard. Under these conditions, the maximum and minimum values of the diyne group wavenumber were determined to be 2221 and 2210 cm^–1^ when the pH was fixed at 5.5 and 7.5, respectively. Furthermore, this compound was tested on cells treated with the apoptosis-triggering drug etoposide. Cytosolic acidification has been observed during apoptosis, and the new sensor tracked the decrease in pH over time in response to etoposide treatment.^92^

Recently *in vitro* and *ex vivo* SRS microscopy has been receiving considerable attention as a new diagnostic tool. Quantitative assessment of liver steatosis in nonalcoholic steatohepatitis (NASH) was achieved with SRS imaging of lipids, proteins, and DNA at 2850, 2930, and 2960 cm^–1^, respectively. The results revealed that there was no significant difference between the numbers of lipid droplets in the control samples and the pathologically altered samples, while the average size of lipid droplets in the tissue was a discriminating factor. Importantly, hematoxylin and eosin or ORO staining failed to detect microvesicular steatosis that can be a hallmark of early-stage disease.^109^ Additionally, brain tumor detection was possible in fresh biopsy samples,^110,111^ opening the prospect for application of SRS as an intraoperative diagnostic tool.^112^ The potential of SRS over routine histological staining, such as analysis of fresh samples, short time of image acquisition, and comparable results for different tissue types,^113^ leads to the conclusion that this technique would be a promising tool in a clinically relevant setting of intraoperative diagnosis.

## 5 Summary and Discussion

Over the past decade, Raman imaging has proven to be useful for detecting biochemical changes in ED in various models of cardiovascular diseases *ex vivo*, *in vivo*, as well as in endothelial cell models *in vitro* mimicking endothelial pathology. However, it has been challenging to study the primary cellular processes occurring in specific endothelial organelles using this technique. Furthermore, ED is related to changes occurring in the mitochondria, nucleus, endoplasmic reticulum, and lysosomes, hence, better understanding of their relative contributions to ED is needed. The nucleus or mitochondria can be imaged *via* Raman microscopy because of the characteristic signals from DNA and cytochrome *c*, respectively, but they are not always so specifically related to these organelles. Nonetheless, there is no Raman marker for small organelles (*e.g.*, lysosomes). Interestingly, there have been a vast number of studies focused on the development of Raman reporters designed to enhance the performance of Raman imaging with a special focus on SRS-based approaches that could be specifically designed to study a given organelle associated with the development of ED. Each reporter possesses in its structure the “targeting moiety” or “sensing group” that is responsible for directing it into a certain organelle along with the molecular reporting group that is characterized by a unique, well-separated Raman signal. The actual subcellular localization of designed Raman reporters is frequently estimated in reference to fluorescence microscopy. Thus, the application of Raman reporters seems to be a promising approach in achieving better sensitivity of Raman imaging (both spontaneous and stimulated) and offering an attractive possibility to identify and image biochemical alterations at the subcellular level in a given organelle. Although there are a few candidates for Raman reporters targeting the nucleus or mitochondria, the propositions of Raman reporters for ER are rather scarce. Still, the specificity of Raman reporters to given organelles represents their limitation as well as their ability to alter the organelles’ function. For example, MitoBADY designed to target mitochondria may accumulate in, *e.g*., lipids, while positively charged mitochondrial probes can alter the mitochondrial membrane potential.

An important asset of Raman-based studies, in particular SRS, described in this review, is the ability of this methodology to visualize the uptake and intracellular distribution of labeled glucose, cholesterol, and other bioenergetic substrates or drugs. Chemical modification of such molecules with deuterium labeling or coupling with Raman reporting moiety opens new avenue in labeled Raman microscopy studies in biomedicine.

In summary, in this review we aimed to provide evidence that Raman reporters can be used not only to target the subcellular structures or follow the fate of labeled molecules but also to monitor particular subcellular processes, which could substantially expand our understanding of the biochemical alteration of ED at the subcellular level. The development and optimization of Raman probes specifically designed to visualize alterations in biochemical content on the subcellular level will surely bring important novel opportunities. Since SRS in combination with the use of Raman reporters has not yet been used to study ED, that approach can be considered as an exciting new experimental method for in-depth characterization of biochemical alterations of endothelial phenotype.

## Abbreviations Used

ED: endothelial dysfunction
ER: endoplasmic reticulum
ROS: reactive oxygen species
SRS: stimulated Raman scattering
epr-SRS: electronic preresonance stimulated Raman scattering
UPR: unfolded protein response

## References

[ref1] Rajendran P.; Rengarajan T.; Thangavel J.; Nishigaki Y.; Sakthisekaran D.; Sethi G.; Nishigaki I. The Vascular Endothelium and Human Diseases. Int. J. Biol. Sci. 2013, 9 (10), 1057–1069. 10.7150/ijbs.7502. 24250251 PMC3831119

[ref2] Battson M. L.; Lee D. M.; Gentile C. L. Endoplasmic Reticulum Stress and the Development of Endothelial Dysfunction. Am. J. Physiol. Circ. Physiol. 2017, 312 (3), H355–H367. 10.1152/ajpheart.00437.2016. 27923788

[ref3] Maamoun H.; Abdelsalam S. S.; Zeidan A.; Korashy H. M.; Agouni A. Endoplasmic Reticulum Stress: A Critical Molecular Driver of Endothelial Dysfunction and Cardiovascular Disturbances Associated with Diabetes. Int. J. Mol. Sci. 2019, 20 (7), 1658 10.3390/ijms20071658. PMC6480154 30987118

[ref4] Pober J. S.; Sessa W. C. Evolving Functions of Endothelial Cells in Inflammation. Nat. Rev. Immunol. 2007, 7 (10), 803–815. 10.1038/nri2171. 17893694

[ref5] Cerutti C.; Ridley A. J. Endothelial Cell-Cell Adhesion and Signaling. Exp. Cell Res. 2017, 358 (1), 31–38. 10.1016/j.yexcr.2017.06.003. 28602626 PMC5700119

[ref6] Mittal M.; Siddiqui M. R.; Tran K.; Reddy S. P.; Malik A. B. Reactive Oxygen Species in Inflammation and Tissue Injury. Antioxid. Redox Signaling 2014, 20 (7), 1126–1167. 10.1089/ars.2012.5149. PMC3929010 23991888

[ref7] Mackman N.; Tilley R. E.; Key N. S. Role of the Extrinsic Pathway of Blood Coagulation in Hemostasis and Thrombosis. Arterioscler., Thromb., Vasc. Biol. 2007, 27 (8), 1687–1693. 10.1161/ATVBAHA.107.141911. 17556654

[ref8] Daiber A.; Chlopicki S. Revisiting Pharmacology of Oxidative Stress and Endothelial Dysfunction in Cardiovascular Disease: Evidence for Redox-Based Therapies. Free Radical Biol. Med. 2020, 157, 15–37. 10.1016/j.freeradbiomed.2020.02.026. 32131026

[ref9] Vita J.; Keaney J. Endothelial Function: A Barometer for Cardiovascular Risk?. Circulation 2002, 106, 640–642. 10.1161/01.CIR.0000028581.07992.56. 12163419

[ref10] Chlopicki S. Perspectives in Pharmacology of Endothelium: From Bench to Bedside. Pharmacol. Rep. 2015, 67, vi–ix. 10.1016/j.pharep.2015.08.005. 26321287

[ref11] Smith R.; Wright K. L.; Ashton L. Raman Spectroscopy: An Evolving Technique for Live Cell Studies. Analyst 2016, 141 (12), 3590–3600. 10.1039/C6AN00152A. 27072718

[ref12] Barańska M.; Małek K.; Bukowska J.; Skirlińska A.; Lipska K.; Likowska A.; Czamara K.; Dybaś J.; Głogowska M.; Jaworska A.; Kaczor A.; Kochan K. Fundamentals of Raman Scattering Spectroscopy. In Vibrational Spectroscopy: From Theory to Practice; Malek K., Ed.; Wydawnictwo Naukowe PWN: Warsaw, 2016; pp 19–25.

[ref13] Dietzek B.; Cialla D.; Schmitt M.; Popp J. Introduction to the Fundamentals of Raman Spectroscopy. Springer Ser. Surf. Sci. 2018, 66, 47–68. 10.1007/978-3-319-75380-5_3.

[ref14] Robert B. Resonance Raman Spectroscopy. Photosynth. Res. 2009, 101 (2–3), 147–155. 10.1007/s11120-009-9440-4. 19568956

[ref15] Hu F.; Brucks S. D.; Lambert T. H.; Campos L. M.; Min W. Stimulated Raman Scattering of Polymer Nanoparticles for Multiplexed Live-Cell Imaging. Chem. Commun. 2017, 53 (46), 6187–6190. 10.1039/C7CC01860F. PMC5623589 28474031

[ref16] Zhang D.; Slipchenko M. N.; Cheng J.-X. Highly Sensitive Vibrational Imaging by Femtosecond Pulse Stimulated Raman Loss. J. Phys. Chem. Lett. 2011, 2 (11), 1248–1253. 10.1021/jz200516n. 21731798 PMC3124560

[ref17] Wei L.; Min W. Electronic Preresonance Stimulated Raman Scattering Microscopy. J. Phys. Chem. Lett. 2018, 9 (15), 4294–4301. 10.1021/acs.jpclett.8b00204. 30001137 PMC6077771

[ref18] Baranska M.; Kaczor A.; Malek K.; Jaworska A.; Majzner K.; Staniszewska-Slezak E.; Pacia M. Z.; Zajac G.; Dybas J.; Wiercigroch E. Raman Microscopy as a Novel Tool to Detect Endothelial Dysfunction. Pharmacol. Rep. 2015, 67 (4), 736–743. 10.1016/j.pharep.2015.03.015. 26321275

[ref19] Hadi H. A. R.; Al Suwaidi J. Endothelial Dysfunction in Diabetes Mellitus. Vasc. Health Risk Manage. 2007, 3 (6), 853–876. PMC2350146 18200806

[ref20] Pilarczyk M.; Rygula A.; Kaczor A.; Mateuszuk L.; Maślak E.; Chlopicki S.; Baranska M. A Novel Approach to Investigate Vascular Wall in 3D: Combined Raman Spectroscopy and Atomic Force Microscopy for Aorta En Face Imaging. Vib. Spectrosc. 2014, 75, 39–44. 10.1016/j.vibspec.2014.09.004.

[ref21] Pilarczyk M.; Mateuszuk L.; Rygula A.; Kepczynski M.; Chlopicki S.; Baranska M.; Kaczor A. Endothelium in Spots – High-Content Imaging of Lipid Rafts Clusters in Db/Db Mice. PLoS One 2014, 9 (8), e106065 10.1371/journal.pone.0106065. 25166908 PMC4148353

[ref22] Pacia M. Z.; Mateuszuk L.; Buczek E.; Chlopicki S.; Blazejczyk A.; Wietrzyk J.; Baranska M.; Kaczor A. Rapid Biochemical Profiling of Endothelial Dysfunction in Diabetes, Hypertension and Cancer Metastasis by Hierarchical Cluster Analysis of Raman Spectra. J. Raman Spectrosc. 2016, 47 (11), 1310–1317. 10.1002/jrs.4965.

[ref23] Hermann M.; Flammer A.; Lüscher T. F. Nitric Oxide in Hypertension. J. Clin. Hypertens. 2006, 8 (12 Suppl. 4), 17–29. 10.1111/j.1524-6175.2006.06032.x. PMC8109558 17170603

[ref24] Muldowney J. A. S.; Davis S. N.; Vaughan D. E.; Brown N. J. NO Synthase Inhibition Increases Aldosterone in Humans. Hypertension 2004, 44 (5), 739–745. 10.1161/01.HYP.0000143852.48258.f1. 15381675

[ref25] Pacia M. Z.; Mateuszuk L.; Chlopicki S.; Baranska M.; Kaczor A. Biochemical Changes of the Endothelium in the Murine Model of NO-Deficient Hypertension. Analyst 2015, 140 (7), 2178–2184. 10.1039/C4AN01870B. 25502217

[ref26] Lee Y. T.; Lin H. Y.; Chan Y. W. F.; Li K. H. C.; To O. T. L.; Yan B. P.; Liu T.; Li G.; Wong W. T.; Keung W.; Tse G. Mouse Models of Atherosclerosis: A Historical Perspective and Recent Advances. Lipids Health Dis. 2017, 16 (1), 12 10.1186/s12944-016-0402-5. 28095860 PMC5240327

[ref27] Maase M.; Rygula A.; Pacia M. Z.; Proniewski B.; Mateuszuk L.; Sternak M.; Kaczor A.; Chlopicki S.; Kusche-Vihrog K. Combined Raman- and AFM-Based Detection of Biochemical and Nanomechanical Features of Endothelial Dysfunction in Aorta Isolated from ApoE/LDLR–/– Mice. Nanomedicine 2019, 16, 97–105. 10.1016/j.nano.2018.11.014. 30550804

[ref28] Marzec K. M.; Rygula A.; Gasior-Glogowska M.; Kochan K.; Czamara K.; Bulat K.; Malek K.; Kaczor A.; Baranska M. Vascular Diseases Investigated Ex Vivo by Using Raman, FT-IR and Complementary Methods. Pharmacol. Rep. 2015, 67 (4), 744–750. 10.1016/j.pharep.2015.05.001. 26321276

[ref29] Cunnington C.; Channon K. M. Tetrahydrobiopterin: Pleiotropic Roles in Cardiovascular Pathophysiology. Heart 2010, 96 (23), 1872–1877. 10.1136/hrt.2009.180430. 20837663

[ref30] Rygula A.; Pacia M. Z.; Mateuszuk L.; Kaczor A.; Kostogrys R. B.; Chlopicki S.; Baranska M. Identification of a Biochemical Marker for Endothelial Dysfunction Using Raman Spectroscopy. Analyst 2015, 140 (7), 2185–2189. 10.1039/C4AN01998A. 25664353

[ref31] Bar A.; Olkowicz M.; Tyrankiewicz U.; Kus E.; Jasinski K.; Smolenski R. T.; Skorka T.; Chlopicki S. Functional and Biochemical Endothelial Profiling In Vivo in a Murine Model of Endothelial Dysfunction; Comparison of Effects of 1-Methylnicotinamide and Angiotensin-Converting Enzyme Inhibitor. Front. Pharmacol. 2017, 8, 183 10.3389/fphar.2017.00183. 28443021 PMC5385379

[ref32] Mateuszuk L.; Jasztal A.; Maslak E.; Gasior-Glogowska M.; Baranska M.; Sitek B.; Kostogrys R.; Zakrzewska A.; Kij A.; Walczak M.; Chlopicki S. Antiatherosclerotic Effects of 1-Methylnicotinamide in Apolipoprotein E/Low-Density Lipoprotein Receptor-Deficient Mice: A Comparison with Nicotinic Acid. J. Pharmacol. Exp. Ther. 2016, 356 (2), 514–524. 10.1124/jpet.115.228643. 26631491 PMC6047228

[ref33] Palonpon A.; Sodeoka M.; Fujita K. Molecular Imaging of Live Cells by Raman Microscopy. Curr. Opin. Chem. Biol. 2013, 17 (4), 708–715. 10.1016/j.cbpa.2013.05.021. 23773582

[ref34] Yamakoshi H.; Dodo K.; Palonpon A.; Ando J.; Fujita K.; Kawata S.; Sodeoka M. Alkyne-Tag Raman Imaging for Visualization of Mobile Small Molecules in Live Cells. J. Am. Chem. Soc. 2012, 134 (51), 20681–20689. 10.1021/ja308529n. 23198907

[ref35] Zhao Z.; Shen Y.; Hu F.; Min W. Applications of Vibrational Tags in Biological Imaging by Raman Microscopy. Analyst 2017, 142 (21), 4018–4029. 10.1039/C7AN01001J. 28875184 PMC5674523

[ref36] Groschner L. N.; Waldeck-Weiermair M.; Malli R.; Graier W. F. Endothelial Mitochondria—Less Respiration, More Integration. Pfluegers Arch. 2012, 464 (1), 63–76. 10.1007/s00424-012-1085-z. 22382745 PMC3387498

[ref37] Kluge M.; Fetterman J.; Vita J. Mitochondria and Endothelial Function. Circ. Res. 2013, 112, 1171–1188. 10.1161/CIRCRESAHA.111.300233. 23580773 PMC3700369

[ref38] Murphy E.; Ardehali H.; Balaban R. S.; DiLisa F.; Dorn G. W.; Kitsis R. N.; Otsu K.; Ping P.; Rizzuto R.; Sack M. N.; Wallace D.; Youle R. J. Mitochondrial Function, Biology, and Role in Disease. Circ. Res. 2016, 118 (12), 1960–1991. 10.1161/RES.0000000000000104. 27126807 PMC6398603

[ref39] Du X. L.; Edelstein D.; Dimmeler S.; Ju Q.; Sui C.; Brownlee M. Hyperglycemia Inhibits Endothelial Nitric Oxide Synthase Activity by Posttranslational Modification at the Akt Site. J. Clin. Invest. 2001, 108 (9), 1341–1348. 10.1172/JCI11235. 11696579 PMC209429

[ref40] Du X.; Matsumura T.; Edelstein D.; Rossetti L.; Zsengellér Z.; Szabó C.; Brownlee M. Inhibition of GAPDH Activity by Poly(ADP-Ribose) Polymerase Activates Three Major Pathways of Hyperglycemic Damage in Endothelial Cells. J. Clin. Invest. 2003, 112 (7), 1049–1057. 10.1172/JCI18127. 14523042 PMC198524

[ref41] Hammes H.-P.; Du X.; Edelstein D.; Taguchi T.; Matsumura T.; Ju Q.; Lin J.; Bierhaus A.; Nawroth P.; Hannak D.; Neumaier M.; Bergfeld R.; Giardino I.; Brownlee M. Benfotiamine Blocks Three Major Pathways of Hyperglycemic Damage and Prevents Experimental Diabetic Retinopathy. Nat. Med. 2003, 9, 294–299. 10.1038/nm834. 12592403

[ref42] Dikalov S. I.; Nazarewicz R. R.; Bikineyeva A.; Hilenski L.; Lassègue B.; Griendling K. K.; Harrison D. G.; Dikalova A. E. Nox2-Induced Production of Mitochondrial Superoxide in Angiotensin II-Mediated Endothelial Oxidative Stress and Hypertension. Antioxid. Redox Signaling 2014, 20 (2), 281–294. 10.1089/ars.2012.4918. PMC3887459 24053613

[ref43] Doughan A. K.; Harrison D. G.; Dikalov S. I. Molecular Mechanisms of Angiotensin II-Mediated Mitochondrial Dysfunction. Circ. Res. 2008, 102 (4), 488–496. 10.1161/CIRCRESAHA.107.162800. 18096818

[ref44] Bunney P. E.; Zink A. N.; Holm A. A.; Billington C. J.; Kotz C. M. Orexin Activation Counteracts Decreases in Nonexercise Activity Thermogenesis (NEAT) Caused by High-Fat Diet. Physiol. Behav. 2017, 176, 139–148. 10.1016/j.physbeh.2017.03.040. 28363838 PMC5510739

[ref45] Okada M.; Smith N. I.; Palonpon A. F.; Endo H.; Kawata S.; Sodeoka M.; Fujita K. Label-Free Raman Observation of Cytochrome *c* Dynamics during Apoptosis. Proc. Natl. Acad. Sci. U. S. A. 2012, 109 (1), 28–32. 10.1073/pnas.1107524108. 22184220 PMC3252932

[ref46] Morimoto T.; Chiu L.; Kanda H.; Kawagoe H.; Ozawa T.; Nakamura M.; Nishida K.; Fujita K.; Fujikado T. Using Redox-Sensitive Mitochondrial Cytochrome Raman Bands for Label-Free Detection of Mitochondrial Dysfunction. Analyst 2019, 144 (8), 2531–2540. 10.1039/C8AN02213E. 30839952

[ref47] Bik E.; Mateuszuk L.; Stojak M.; Chlopicki S.; Baranska M.; Majzner K. Menadione-Induced Endothelial Inflammation Detected by Raman Spectroscopy. Biochim. Biophys. Acta, Mol. Cell Res. 2021, 1868 (2), 118911 10.1016/j.bbamcr.2020.118911. 33227312

[ref48] Yamakoshi H.; Palonpon A.; Dodo K.; Ando J.; Kawata S.; Fujita K.; Sodeoka M. A Sensitive and Specific Raman Probe Based on Bisarylbutadiyne for Live Cell Imaging of Mitochondria. Bioorg. Med. Chem. Lett. 2015, 25 (3), 664–667. 10.1016/j.bmcl.2014.11.080. 25522818

[ref49] Bae K.; Zheng W.; Ma Y.; Huang Z. Real-Time Monitoring of Pharmacokinetics of Mitochondria-Targeting Molecules in Live Cells with Bioorthogonal Hyperspectral Stimulated Raman Scattering Microscopy. Anal. Chem. 2020, 92 (1), 740–748. 10.1021/acs.analchem.9b02838. 31750649

[ref50] Zielonka J.; Joseph J.; Sikora A.; Hardy M.; Ouari O.; Vasquez-Vivar J.; Cheng G.; Lopez M.; Kalyanaraman B. Mitochondria-Targeted Triphenylphosphonium-Based Compounds: Syntheses, Mechanisms of Action, and Therapeutic and Diagnostic Applications. Chem. Rev. 2017, 117 (15), 10043–10120. 10.1021/acs.chemrev.7b00042. 28654243 PMC5611849

[ref51] Gao P.; Pan W.; Li N.; Tang B. Fluorescent Probes for Organelle-Targeted Bioactive Species Imaging. Chem. Sci. 2019, 10 (24), 6035–6071. 10.1039/C9SC01652J. 31360411 PMC6585876

[ref52] Li Y.; Heo J.; Lim C. K.; Pliss A.; Kachynski A. V.; Kuzmin A. N.; Kim S.; Prasad P. N. Organelle Specific Imaging in Live Cells and Immuno-Labeling Using Resonance Raman Probe. Biomaterials 2015, 53, 25–31. 10.1016/j.biomaterials.2015.02.056. 25890703

[ref53] Hu F.; Zeng C.; Long R.; Miao Y.; Wei L.; Xu Q.; Min W. Supermultiplexed Optical Imaging and Barcoding with Engineered Polyynes. Nat. Methods 2018, 15 (3), 194–200. 10.1038/nmeth.4578. 29334378 PMC5831481

[ref54] Agemy L.; Kotamraju V. R.; Friedmann-Morvinski D.; Sharma S.; Sugahara K. N.; Ruoslahti E. Proapoptotic Peptide-Mediated Cancer Therapy Targeted to Cell Surface P32. Mol. Ther. 2013, 21 (12), 2195–2204. 10.1038/mt.2013.191. 23959073 PMC3863797

[ref55] Tian S.; Li H.; Li Z.; Tang H.; Yin M.; Chen Y.; Wang S.; Gao Y.; Yang X.; Meng F.; Lauher J. W.; Wang P.; Luo L. Polydiacetylene-Based Ultrastrong Bioorthogonal Raman Probes for Targeted Live-Cell Raman Imaging. Nat. Commun. 2020, 11 (1), 81 10.1038/s41467-019-13784-0. 31900403 PMC6941979

[ref56] Wei L.; Chen Z.; Shi L.; Long R.; Anzalone A. V.; Zhang L.; Hu F.; Yuste R.; Cornish V. W.; Min W. Super-Multiplex Vibrational Imaging. Nature 2017, 544, 465 10.1038/nature22051. 28424513 PMC5939925

[ref57] Fiorentino T. V.; Procopio T.; Mancuso E.; Arcidiacono G. P.; Andreozzi F.; Arturi F.; Sciacqua A.; Perticone F.; Hribal M. L.; Sesti G. SRT1720 Counteracts Glucosamine-Induced Endoplasmic Reticulum Stress and Endothelial Dysfunction. Cardiovasc. Res. 2015, 107 (2), 295–306. 10.1093/cvr/cvv169. 26038299

[ref58] Luchetti F.; Crinelli R.; Cesarini E.; Canonico B.; Guidi L.; Zerbinati C.; Di Sario G.; Zamai L.; Magnani M.; Papa S.; Iuliano L. Endothelial Cells, Endoplasmic Reticulum Stress and Oxysterols. Redox Biol. 2017, 13, 581–587. 10.1016/j.redox.2017.07.014. 28783588 PMC5545768

[ref59] Visioli F.; Artaria C. Astaxanthin in Cardiovascular Health and Disease: Mechanisms of Action, Therapeutic Merits, and Knowledge Gaps. Food Funct. 2017, 8 (1), 39–63. 10.1039/C6FO01721E. 27924978

[ref60] Bik E.; Mielniczek N.; Jarosz M.; Denbigh J.; Budzynska R.; Baranska M.; Majzner K. Tunicamycin Induced Endoplasmic Reticulum Changes in Endothelial Cells Investigated in Vitro by Confocal Raman Imaging. Analyst 2019, 144 (22), 6561–6569. 10.1039/C9AN01456J. 31576836

[ref61] Czamara K.; Petko F.; Baranska M.; Kaczor A. Raman Microscopy at the Subcellular Level: A Study on Early Apoptosis in Endothelial Cells Induced by Fas Ligand and Cycloheximide. Analyst 2016, 141 (4), 1390–1397. 10.1039/C5AN02202A. 26765153

[ref62] Wei L.; Yu Y.; Shen Y.; Wang M. C.; Min W. Vibrational Imaging of Newly Synthesized Proteins in Live Cells by Stimulated Raman Scattering Microscopy. Proc. Natl. Acad. Sci. U. S. A. 2013, 110 (28), 11226–11231. 10.1073/pnas.1303768110. 23798434 PMC3710790

[ref63] Wei L.; Shen Y.; Xu F.; Hu F.; Harrington J. K.; Targoff K. L.; Min W. Imaging Complex Protein Metabolism in Live Organisms by Stimulated Raman Scattering Microscopy with Isotope Labeling. ACS Chem. Biol. 2015, 10 (3), 901–908. 10.1021/cb500787b. 25560305 PMC4610303

[ref64] Shen Y.; Xu F.; Wei L.; Hu F.; Min W. Live-Cell Quantitative Imaging of Proteome Degradation by Stimulated Raman Scattering. Angew. Chem., Int. Ed. 2014, 53 (22), 5596–5599. 10.1002/anie.201310725. PMC4231775 24737659

[ref65] Miao K.; Wei L. Live-Cell Imaging and Quantification of PolyQ Aggregates by Stimulated Raman Scattering of Selective Deuterium Labeling. ACS Cent. Sci. 2020, 6 (4), 478–486. 10.1021/acscentsci.9b01196. 32341997 PMC7181319

[ref66] Wei L.; Hu F.; Shen Y.; Chen Z.; Yu Y.; Lin C.-C.; Wang M. C.; Min W. Live-Cell Imaging of Alkyne-Tagged Small Biomolecules by Stimulated Raman Scattering. Nat. Methods 2014, 11, 410 10.1038/nmeth.2878. 24584195 PMC4040164

[ref67] Hong S.; Chen T.; Zhu Y.; Li A.; Huang Y.; Chen X. Live-Cell Stimulated Raman Scattering Imaging of Alkyne-Tagged Biomolecules. Angew. Chem., Int. Ed. 2014, 53 (23), 5827–5831. 10.1002/anie.201400328. 24753329

[ref68] Berry D.; Mader E.; Lee T. K.; Woebken D.; Wang Y.; Zhu D.; Palatinszky M.; Schintlmeister A.; Schmid M. C.; Hanson B. T.; Shterzer N.; Mizrahi I.; Rauch I.; Decker T.; Bocklitz T.; Popp J.; Gibson C. M.; Fowler P. W.; Huang W. E.; Wagner M. Tracking Heavy Water (D_2_O) Incorporation for Identifying and Sorting Active Microbial Cells. Proc. Natl. Acad. Sci. U. S. A. 2015, 112 (2), E194–E203. 10.1073/pnas.1420406112. 25550518 PMC4299247

[ref69] Tao Y.; Wang Y.; Huang S.; Zhu P.; Huang W. E.; Ling J.; Xu J. Metabolic-Activity-Based Assessment of Antimicrobial Effects by D2O-Labeled Single-Cell Raman Microspectroscopy. Anal. Chem. 2017, 89 (7), 4108–4115. 10.1021/acs.analchem.6b05051. 28282113

[ref70] Shi L.; Zheng C.; Shen Y.; Chen Z.; Silveira E. S.; Zhang L.; Wei M.; Liu C.; de Sena-Tomas C.; Targoff K.; Min W. Optical Imaging of Metabolic Dynamics in Animals. Nat. Commun. 2018, 9 (1), 2995 10.1038/s41467-018-05401-3. 30082908 PMC6079036

[ref71] Shen Y.; Zhao Z.; Zhang L.; Shi L.; Shahriar S.; Chan R. B.; Di Paolo G.; Min W. Metabolic Activity Induces Membrane Phase Separation in Endoplasmic Reticulum. Proc. Natl. Acad. Sci. U. S. A. 2017, 114 (51), 13394–13399. 10.1073/pnas.1712555114. 29196526 PMC5754785

[ref72] Duer M.; Cobb A. M.; Shanahan C. M. DNA Damage Response. Arterioscler., Thromb., Vasc. Biol. 2020, 40, e193–e202. 10.1161/ATVBAHA.120.313792. 32404005

[ref73] Notingher I. Raman Spectroscopy Cell-Based Biosensors. Sensors 2007, 7 (8), 1343–1358. 10.3390/s7081343.

[ref74] Salic A.; Mitchison T. J. A Chemical Method for Fast and Sensitive Detection of DNA Synthesis in Vivo. Proc. Natl. Acad. Sci. U. S. A. 2008, 105 (7), 2415–2420. 10.1073/pnas.0712168105. 18272492 PMC2268151

[ref75] Yamakoshi H.; Dodo K.; Okada M.; Ando J.; Palonpon A.; Fujita K.; Kawata S.; Sodeoka M. Imaging of EdU, an Alkyne-Tagged Cell Proliferation Probe, by Raman Microscopy. J. Am. Chem. Soc. 2011, 133 (16), 6102–6105. 10.1021/ja108404p. 21443184

[ref76] Chen Z.; Paley D. W.; Wei L.; Weisman A. L.; Friesner R. A.; Nuckolls C.; Min W. Multicolor Live-Cell Chemical Imaging by Isotopically Edited Alkyne Vibrational Palette. J. Am. Chem. Soc. 2014, 136 (22), 8027–8033. 10.1021/ja502706q. 24849912 PMC4063185

[ref77] Zhang J.; Yan S.; He Z.; Ding C.; Zhai T.; Chen Y.; Li H.; Yang G.; Zhou X.; Wang P. Small Unnatural Amino Acid Carried Raman Tag for Molecular Imaging of Genetically Targeted Proteins. J. Phys. Chem. Lett. 2018, 9 (16), 4679–4685. 10.1021/acs.jpclett.8b01991. 30067370

[ref78] Jiang F. Autophagy in Vascular Endothelial Cells. Clin. Exp. Pharmacol. Physiol. 2016, 43 (11), 1021–1028. 10.1111/1440-1681.12649. 27558982

[ref79] Schaaf M. B.; Houbaert D.; Meçe O.; Agostinis P. Autophagy in Endothelial Cells and Tumor Angiogenesis. Cell Death Differ. 2019, 26 (4), 665–679. 10.1038/s41418-019-0287-8. 30692642 PMC6460396

[ref80] Nussenzweig S. C.; Verma S.; Finkel T. The Role of Autophagy in Vascular Biology. Circ. Res. 2015, 116 (3), 480–488. 10.1161/CIRCRESAHA.116.303805. 25634971 PMC4313568

[ref81] Bao J.-X.; Xia M.; Poklis J. L.; Han W.-Q.; Brimson C.; Li P.-L. Triggering Role of Acid Sphingomyelinase in Endothelial Lysosome-Membrane Fusion and Dysfunction in Coronary Arteries. Am. J. Physiol. Circ. Physiol. 2010, 298 (3), H992–H1002. 10.1152/ajpheart.00958.2009. PMC2838547 20061541

[ref82] Bao J.-X.; Chang H.; Lv Y.-G.; Yu J.-W.; Bai Y.-G.; Liu H.; Cai Y.; Wang L.; Ma J.; Chang Y.-M. Lysosome-Membrane Fusion Mediated Superoxide Production in Hyperglycaemia-Induced Endothelial Dysfunction. PLoS One 2012, 7 (1), e30387 10.1371/journal.pone.0030387. 22253932 PMC3257261

[ref83] Li W.; Ghosh M.; Eftekhari S.; Yuan X.-M. Lipid Accumulation and Lysosomal Pathways Contribute to Dysfunction and Apoptosis of Human Endothelial Cells Caused by 7-Oxysterols. Biochem. Biophys. Res. Commun. 2011, 409 (4), 711–716. 10.1016/j.bbrc.2011.05.071. 21621514

[ref84] Kuzmin A. N.; Pliss A.; Lim C. K.; Heo J.; Kim S.; Rzhevskii A.; Gu B.; Yong K. T.; Wen S.; Prasad P. N. Resonance Raman Probes for Organelle-Specific Labeling in Live Cells. Sci. Rep. 2016, 6 (1), 28483 10.1038/srep28483. 27339882 PMC4919686

[ref85] Asai T.; Liu H.; Ozeki Y.; Sato S.; Hayashi T.; Nakamura H. Imaging of Cellular Uptake of Boron Cluster Compound by Stimulated Raman Scattering Microscopy. Appl. Phys. Express 2019, 12 (11), 112004 10.7567/1882-0786/ab4a5d.

[ref86] Long R.; Zhang L.; Shi L.; Shen Y.; Hu F.; Zeng C.; Min W. Two-Color Vibrational Imaging of Glucose Metabolism Using Stimulated Raman Scattering. Chem. Commun. 2018, 54 (2), 152–155. 10.1039/C7CC08217G. PMC5764084 29218356

[ref87] Tipping W. J.; Lee M.; Serrels A.; Brunton V. G.; Hulme A. N. Imaging Drug Uptake by Bioorthogonal Stimulated Raman Scattering Microscopy. Chem. Sci. 2017, 8 (8), 5606–5615. 10.1039/C7SC01837A. 30155229 PMC6103005

[ref88] Sepp K.; Lee M.; Bluntzer M. T. J.; Helgason G. V.; Hulme A. N.; Brunton V. G. Utilizing Stimulated Raman Scattering Microscopy To Study Intracellular Distribution of Label-Free Ponatinib in Live Cells. J. Med. Chem. 2020, 63 (5), 2028–2034. 10.1021/acs.jmedchem.9b01546. 31829628 PMC7073915

[ref89] Fujioka H.; Shou J.; Kojima R.; Urano Y.; Ozeki Y.; Kamiya M. Multicolor Activatable Raman Probes for Simultaneous Detection of Plural Enzyme Activities. J. Am. Chem. Soc. 2020, 142 (49), 20701–20707. 10.1021/jacs.0c09200. 33225696

[ref90] Aljakouch K.; Lechtonen T.; Yosef H. K.; Hammoud M. K.; Alsaidi W.; Kötting C.; Mügge C.; Kourist R.; El-Mashtoly S. F.; Gerwert K. Raman Microspectroscopic Evidence for the Metabolism of a Tyrosine Kinase Inhibitor, Neratinib, in Cancer Cells. Angew. Chem., Int. Ed. 2018, 57 (24), 7250–7254. 10.1002/anie.201803394. PMC6033014 29645336

[ref91] El-Mashtoly S. F.; Petersen D.; Yosef H. K.; Mosig A.; Reinacher-Schick A.; Kötting C.; Gerwert K. Label-Free Imaging of Drug Distribution and Metabolism in Colon Cancer Cells by Raman Microscopy. Analyst 2014, 139 (5), 1155–1161. 10.1039/c3an01993d. 24427772

[ref92] Wilson L. T.; Tipping W. J.; Jamieson L. E.; Wetherill C.; Henley Z.; Faulds K.; Graham D.; Mackay S. P.; Tomkinson N. C. O. A New Class of Ratiometric Small Molecule Intracellular PH Sensors for Raman Microscopy. Analyst 2020, 145 (15), 5289–5298. 10.1039/D0AN00865F. 32672252

[ref93] Hu F.; Chen Z.; Zhang L.; Shen Y.; Wei L.; Min W. Vibrational Imaging of Glucose Uptake Activity in Live Cells and Tissues by Stimulated Raman Scattering. Angew. Chem., Int. Ed. 2015, 54 (34), 9821–9825. 10.1002/anie.201502543. PMC4644272 26207979

[ref94] Lee D.; Du J.; Yu R.; Su Y.; Heath J. R.; Wei L. Visualizing Subcellular Enrichment of Glycogen in Live Cancer Cells by Stimulated Raman Scattering. Anal. Chem. 2020, 92 (19), 13182–13191. 10.1021/acs.analchem.0c02348. 32907318 PMC10676777

[ref95] Zhang L.; Shi L.; Shen Y.; Miao Y.; Wei M.; Qian N.; Liu Y.; Min W. Spectral Tracing of Deuterium for Imaging Glucose Metabolism. Nat. Biomed. Eng. 2019, 3 (5), 402–413. 10.1038/s41551-019-0393-4. 31036888 PMC6599680

[ref96] Pacia M. Z.; Sternak M.; Mateuszuk L.; Stojak M.; Kaczor A.; Chlopicki S. Heterogeneity of Chemical Composition of Lipid Droplets in Endothelial Inflammation and Apoptosis. Biochim. Biophys. Acta, Mol. Cell Res. 2020, 1867 (6), 118681 10.1016/j.bbamcr.2020.118681. 32084444

[ref97] Alfonso-García A.; Pfisterer S. G.; Riezman H.; Ikonen E.; Potma E. O. D38-Cholesterol as a Raman Active Probe for Imaging Intracellular Cholesterol Storage. J. Biomed. Opt. 2016, 21 (6), 061003 10.1117/1.JBO.21.6.061003. PMC4681884 26719944

[ref98] Lee H. J.; Zhang W.; Zhang D.; Yang Y.; Liu B.; Barker E. L.; Buhman K. K.; Slipchenko L. V.; Dai M.; Cheng J.-X. Assessing Cholesterol Storage in Live Cells and C. Elegans by Stimulated Raman Scattering Imaging of Phenyl-Diyne Cholesterol. Sci. Rep. 2015, 5, 7930 10.1038/srep07930. 25608867 PMC4302291

[ref99] Majzner K.; Tott S.; Roussille L.; Deckert V.; Chlopicki S.; Baranska M. Uptake of Fatty Acids by a Single Endothelial Cell Investigated by Raman Spectroscopy Supported by AFM. Analyst 2018, 143 (4), 970–980. 10.1039/C7AN01043E. 29372724

[ref100] Jamieson L. E.; Greaves J.; McLellan J. A.; Munro K. R.; Tomkinson N. C. O.; Chamberlain L. H.; Faulds K.; Graham D. Tracking Intracellular Uptake and Localisation of Alkyne Tagged Fatty Acids Using Raman Spectroscopy. Spectrochim. Acta, Part A 2018, 197, 30–36. 10.1016/j.saa.2018.01.064. PMC5890826 29525355

[ref101] Li X.; Li Y.; Jiang M.; Wu W.; He S.; Chen C.; Qin Z.; Tang B. Z.; Mak H. Y.; Qu J. Y. Quantitative Imaging of Lipid Synthesis and Lipolysis Dynamics in Caenorhabditis Elegans by Stimulated Raman Scattering Microscopy. Anal. Chem. 2019, 91 (3), 2279–2287. 10.1021/acs.analchem.8b04875. 30589537

[ref102] Stiebing C.; Meyer T.; Rimke I.; Matthäus C.; Schmitt M.; Lorkowski S.; Popp J. Real-Time Raman and SRS Imaging of Living Human Macrophages Reveals Cell-to-Cell Heterogeneity and Dynamics of Lipid Uptake. J. Biophotonics 2017, 10 (9), 1217–1226. 10.1002/jbio.201600279. 28164480

[ref103] Czamara K.; Adamczyk A.; Stojak M.; Radwan B.; Baranska M. Astaxanthin as a New Raman Probe for Biosensing of Specific Subcellular Lipidic Structures: Can We Detect Lipids in Cells under Resonance Conditions?. Cell. Mol. Life Sci. 2020, 10.1007/s00018-020-03718-1. PMC8038953 33289850

[ref104] Hu F.; Wei L.; Zheng C.; Shen Y.; Min W. Live-Cell Vibrational Imaging of Choline Metabolites by Stimulated Raman Scattering Coupled with Isotope-Based Metabolic Labeling. Analyst 2014, 139 (10), 2312–2317. 10.1039/C3AN02281A. 24555181 PMC4069604

[ref105] Seidel J.; Miao Y.; Porterfield W.; Cai W.; Zhu X.; Kim S.-J.; Hu F.; Bhattarai-Kline S.; Min W.; Zhang W. Structure–Activity–Distribution Relationship Study of Anti-Cancer Antimycin-Type Depsipeptides. Chem. Commun. 2019, 55 (63), 9379–9382. 10.1039/C9CC03051D. PMC6675640 31317975

[ref106] Fu D.; Zhou J.; Zhu W. S.; Manley P. W.; Wang Y. K.; Hood T.; Wylie A.; Xie X. S. Imaging the Intracellular Distribution of Tyrosine Kinase Inhibitors in Living Cells with Quantitative Hyperspectral Stimulated Raman Scattering. Nat. Chem. 2014, 6 (7), 614–622. 10.1038/nchem.1961. 24950332 PMC4205760

[ref107] Wojcik T.; Szczesny E.; Chlopicki S. Detrimental Effects of Chemotherapeutics and Other Drugs on the Endothelium: A Call for Endothelial Toxicity Profiling. Pharmacol. Rep. 2015, 67 (4), 811–817. 10.1016/j.pharep.2015.03.022. 26321285

[ref108] Manouchehri A.; Kanu E.; Mauro M. J.; Aday A. W.; Lindner J. R.; Moslehi J. Tyrosine Kinase Inhibitors in Leukemia and Cardiovascular Events: From Mechanism to Patient Care. Arterioscler., Thromb., Vasc. Biol. 2020, 40 (2), 301–308. 10.1161/ATVBAHA.119.313353. 31875699 PMC6993877

[ref109] Urasaki Y.; Zhang C.; Cheng J.-X.; Le T. T. Quantitative Assessment of Liver Steatosis and Affected Pathways with Molecular Imaging and Proteomic Profiling. Sci. Rep. 2018, 8 (1), 3606 10.1038/s41598-018-22082-6. 29483581 PMC5826939

[ref110] Shin K. S.; Francis A. T.; Hill A. H.; Laohajaratsang M.; Cimino P. J.; Latimer C. S.; Gonzalez-Cuyar L. F.; Sekhar L. N.; Juric-Sekhar G.; Fu D. Intraoperative Assessment of Skull Base Tumors Using Stimulated Raman Scattering Microscopy. Sci. Rep. 2019, 9 (1), 20392 10.1038/s41598-019-56932-8. 31892723 PMC6938502

[ref111] Ji M.; Lewis S.; Camelo-Piragua S.; Ramkissoon S. H.; Snuderl M.; Venneti S.; Fisher-Hubbard A.; Garrard M.; Fu D.; Wang A. C.; Heth J. A.; Maher C. O.; Sanai N.; Johnson T. D.; Freudiger C. W.; Sagher O.; Xie X. S.; Orringer D. A. Detection of Human Brain Tumor Infiltration with Quantitative Stimulated Raman Scattering Microscopy. Sci. Transl. Med. 2015, 7 (309), 309ra163–309ra163. 10.1126/scitranslmed.aab0195. PMC4900155 26468325

[ref112] Bae K.; Xin L.; Zheng W.; Tang C.; Ang B.-T.; Huang Z. Mapping the Intratumoral Heterogeneity in Glioblastomas with Hyperspectral Stimulated Raman Scattering Microscopy. Anal. Chem. 2021, 93 (4), 2377–2384. 10.1021/acs.analchem.0c04262. 33443405

[ref113] Sarri B.; Poizat F.; Heuke S.; Wojak J.; Franchi F.; Caillol F.; Giovannini M.; Rigneault H. Stimulated Raman Histology: One to One Comparison with Standard Hematoxylin and Eosin Staining. Biomed. Opt. Express 2019, 10 (10), 5378–5384. 10.1364/BOE.10.005378. 31646052 PMC6788596

